# In vivo study to assess fat embolism resulting from the Reamer-Irrigator-Aspirator 2 system compared to a novel aspirator-based concept for intramedullary bone graft harvesting

**DOI:** 10.1007/s00402-024-05220-w

**Published:** 2024-02-17

**Authors:** Markus Laubach, Agathe Bessot, Siamak Saifzadeh, Flavia Medeiros Savi, Frank Hildebrand, Nathalie Bock, Dietmar W. Hutmacher, Jacqui McGovern

**Affiliations:** 1 Australian Research Council (ARC) Training Centre for Multiscale 3D Imaging, Modelling, and Manufacturing (M3D Innovation), Queensland University of Technology, Brisbane, QLD 4000 Australia; 2 Centre for Biomedical Technologies, School of Mechanical, Medical and Process Engineering, Queensland University of Technology, Brisbane, QLD 4059 Australia; 3 Department of Orthopaedics, Trauma and Reconstructive Surgery, RWTH Aachen University Hospital, Pauwelsstraße 30, 52074 Aachen, Germany; 4 Max Planck Queensland Centre for the Materials Science of Extracellular Matrices, Queensland University of Technology, Brisbane, QLD 4000 Australia; 5 Centre for Biomedical Technologies, School of Biomedical Sciences, Faculty of Health, and Translational Research Institute (TRI), Queensland University of Technology (QUT), Brisbane, QLD 4102 Australia; 6 Medical Engineering Research Facility, Queensland University of Technology, Chermside, QLD 4032 Australia; 7 ARC Training Centre for Cell and Tissue Engineering Technologies, Queensland University of Technology (QUT), Brisbane, QLD 4000 Australia; 8 Translational Research Institute (TRI), 37 Kent Street, Woolloongabba, QLD 4102 Australia

**Keywords:** Bone graft, Harvesting, Reaming, Fat embolism

## Abstract

**Introduction:**

Fat embolism (FE) following intramedullary (IM) reaming can cause severe pulmonary complications and sudden death. Recently, a new harvesting concept was introduced in which a novel aspirator is used first for bone marrow (BM) aspiration and then for subsequent aspiration of morselized endosteal bone during sequential reaming (A + R + A). In contrast to the established Reamer-Irrigator-Aspirator (RIA) 2 system, the new A + R + A concept allows for the evacuation of fatty BM prior to reaming. In this study, we hypothesized that the risk of FE, associated coagulopathic reactions and pulmonary FE would be comparable between the RIA 2 system and the A + R + A concept.

**Materials and methods:**

Intramedullary bone graft was harvested from intact femora of 16 Merino sheep (age: 1–2 years) with either the RIA 2 system (n = 8) or the A + R + A concept (n = 8). Fat intravasation was monitored with the Gurd test, coagulopathic response with D-dimer blood level concentration and pulmonary FE with histological evaluation of the lungs.

**Results:**

The total number and average size of intravasated fat particles was similar between groups (*p* = 0.13 and *p* = 0.98, respectively). D-dimer concentration did not significantly increase within 4 h after completion of surgery (RIA 2: *p* = 0.82; A + R + A: *p* = 0.23), with an interaction effect similar between groups (*p* = 0.65). The average lung area covered with fat globules was similar between groups (*p* = 0.17).

**Conclusions:**

The use of the RIA 2 system and the novel A + R + A harvesting concept which consists of BM evacuation followed by sequential IM reaming and aspiration of endosteal bone, resulted in only minor fat intravasation, coagulopathic reactions and pulmonary FE, with no significant differences between the groups. Our results, therefore, suggest that both the RIA 2 system and the new A + R + A concept are comparable technologies in terms of FE-related complications.

**Supplementary Information:**

The online version contains supplementary material available at 10.1007/s00402-024-05220-w.

## Introduction

Harvesting bone graft (BG) from the intramedullary (IM) canal of long bones using the Reamer-Irrigator-Aspirator (RIA) system (Synthes) has become a standard procedure for the treatment of bone defects [1, 2]. To achieve effective BG harvesting, the RIA system applies continuous irrigation and suction during IM reaming of long bones. Recently, a novel harvesting concept aimed at improving the BG collection without the potential washout effect of osteogenic factors in the graft material associated with the RIA system’s irrigation fluid was successfully tested [3, 4]. This novel concept includes the option of initially aspirating the bone marrow (BM) using a novel aspirator prototype (aspirator, A), followed by sequential reaming using standard reamers and collecting BG with the novel aspirator prototype (reamer + aspirator, R + A), which has been shown to have the capacity to preserve the BG’s osteoimmune microenvironment associated with high osteogenic potential (A + R + A concept) [3].

Complications associated with significantly increased IM pressure and subsequent fat intravasation (fat particles entering the bloodstream) have been reported during IM reaming [5]. Fat intravasation causes fat embolism (FE) which has been associated with serious (pulmonary) complications such as adult respiratory distress syndrome, and sudden intraoperative death [6]. In particular, the first stages of IM reaming can be associated with high IM pressure resulting in FE [7, 8]. Thrombogenic fatty BM contents, when expelled as FEs during medullary canal reaming, triggers a coagulopathic reaction. The tissue factors induce thrombin generation [9], activating fibrinolytic cascades. This reaction may cause enlargement of the intravasated fat BM particles, blocking larger pulmonary blood vessels and damaging lung tissue [10, 11]. Additionally, this reaction may lead to disseminated intravascular coagulation, characterized by a widespread hypercoagulable state with widespread clotting, leading to multiple organ dysfunction syndrome [12]. The increased fibrinolytic reactions produces D-dimers [13], making plasma D-dimer level reliable indicator for soft tissue and osseous injuries as well as coagulopathic response due to FE [14, 15].

Various methods, including venting and the RIA irrigation–suction system, have been developed to reduce the IM pressure and FE during reaming. The IM pressure is influenced by the medullary contents’ volume and viscosity, with a more viscous BM increasing pressure and FE risk [16–18]. The RIA system, originally designed to prevent cortical FE in femoral medullary cavity reaming for IM nailing in fracture patients, also facilitates reamer head cooling, marrow emulsification, and debris evacuation [19–21]. Indeed, large animal studies have demonstrated its effectiveness in reducing (fat) embolism [20, 22]. For instance, applying reaming with the RIA system prior to femoral medullary nailing, particularly in cases of (unilateral) pulmonary injury, reduced coagulopathic response and lung injury, as inferred from lower D-dimer blood concentrations compared to standard reaming [20].

Therefore, alternative novel BG harvesting concepts using IM reaming require assessment for perioperative FE. Notably, in a sheep model, effective emptying of the femoral IM canal of the BM using multiple suction ports before reaming of nonfractured bone with conventional reamers was associated with reduced IM pressure and lung embolism [23; 24]. In line with this conceptual approach, the new harvesting concept with a dedicated aspirator allows BM to be evacuated from the IM canal prior to BG harvesting, the overall concept being defined as A + R + A with the use of the aspirator (A, BM evacuation) and reamer + aspirator (R + A, harvesting morselized endosteal bone) [3]. Yet it is crucial to assess its FE capacity to ensure that there are no increased adverse events using this technique. Recently, a second-generation RIA system (RIA 2 system) was launched [25] and works in the same conceptual way as RIA, therefore, used in this study. We thus hypothesized that the novel harvesting concept (A + R + A) is similar to the RIA 2 system (RIA 2) in terms of BM fat intravasation, associated coagulopathic response and pulmonary FE.

## Materials and methods

The study was performed on 16 female Merino sheep (*Ovis aries*, age: 1–2 years; body weight, BW: 41–51 kg) procured from a local farm, and preoperative health checks were performed by a veterinarian as per an established standard protocol [26]. The ARRIVE 2.0 guidelines (Animal Research: Reporting of In Vivo Experiments) [27] were followed. Ethical approval was obtained from the Queensland University of Technology (QUT) Animal Ethics Committee (UAEC) (approval number 2000000593). Preoperative femoral computed tomography (CT) imaging for allocation of the sheep into experimental groups, as per the femoral isthmus diameter with a high-resolution helical acquisition, was performed using a single-source CT (Toshiba Aquilion Lightning™, Tokyo, Japan) under general anaesthesia using isoflurane. Using the open-source medical image viewer Horos (version 3.3.6), the smallest diameter (isthmus) was determined by first measuring the length of the femur as defined from the trochanteric fossa to the condyles. The IM canal diameter at the isthmus was determined at the midway point of the total femur length as described previously in detail [3].

### In vivo procedures and experimental groups

The animals were anaesthetized with propofol (5 mg/kg BW) administered through a central venous catheter (CVC) in the external jugular vein, and then were endotracheally intubated. Anaesthesia was maintained with the animals in the dorsal recumbency position with inhaled isoflurane (2–2.5% in 40% oxygen) combined with intravenous fentanyl (10 μg/kg BW/h) for analgesia. An additional CVC (Quad-Lumen Indwelling Catheter; 8.5 Fr. × 16 cm with Blue FlexTip®; Arrow International, Inc.; PA, USA) was placed using the Seldinger technique in the left external iliac vein (EIV) before surgery. All surgical procedures were performed by the same surgeon (M.L.) as described previously [3]. Briefly, an incision was made 1–2 cm proximal to the greater trochanter, dissection was performed to expose the trochanteric fossa, which was opened under radiographic guidance with a cannulated Ø 10 mm rigid reamer (Stryker Trauma GmbH, Schoenkirchen, Germany) inserted over a K-wire for access to the femoral medullary canal. While avoiding leakage of the BM from the medullary canal, the femoral opening was carefully enlarged using a Ø 11 mm Bixcut fixed-head reamer (Stryker Trauma GmbH, Schoenkirchen, Germany). The reamer heads were replaced after every three experiments in all study groups.

#### RIA 2 group

The RIA 2 system, launched in 2020, including exchangeable reamer heads with the smallest diameter of 10 mm, was used. Application of the RIA 2 system using a guide-wire and a standard drill speed commenced with a reamer head 2 mm narrower than the preoperatively measured diameter of the femoral isthmus, and then size of the reamer head was increased by 1 mm in each of the first two reaming steps. Subsequently, under X-ray surveillance, the size of the reamer head was increased in 0.5 mm increments until approximately 0.5–1 mm of residual cortical bone was visible at the isthmus on the conventional X-ray.

#### A + R + A group

In a first step, using the recently introduced [3] aspirator prototype (A, BM evacuation), BM was aspirated from the medullary cavity. Subsequently, during the second step, reaming was performed using a guide-wire and the Bixcut reamer (modular reamer head kit, Stryker Trauma GmbH, Schoenkirchen, Germany) in sequential steps with increments described above for the RIA 2 group. After each reaming, the cannula of the aspirator was re-inserted into the medullary canal to collect endosteal morselized bone particles. During this procedure, the aspirator was repeatedly advanced and retracted, gently touching the endocortex (R + A, harvesting morselized endosteal bone).

### Assessment and sequalae of bone marrow fat intravasation

**Fig. 1:**
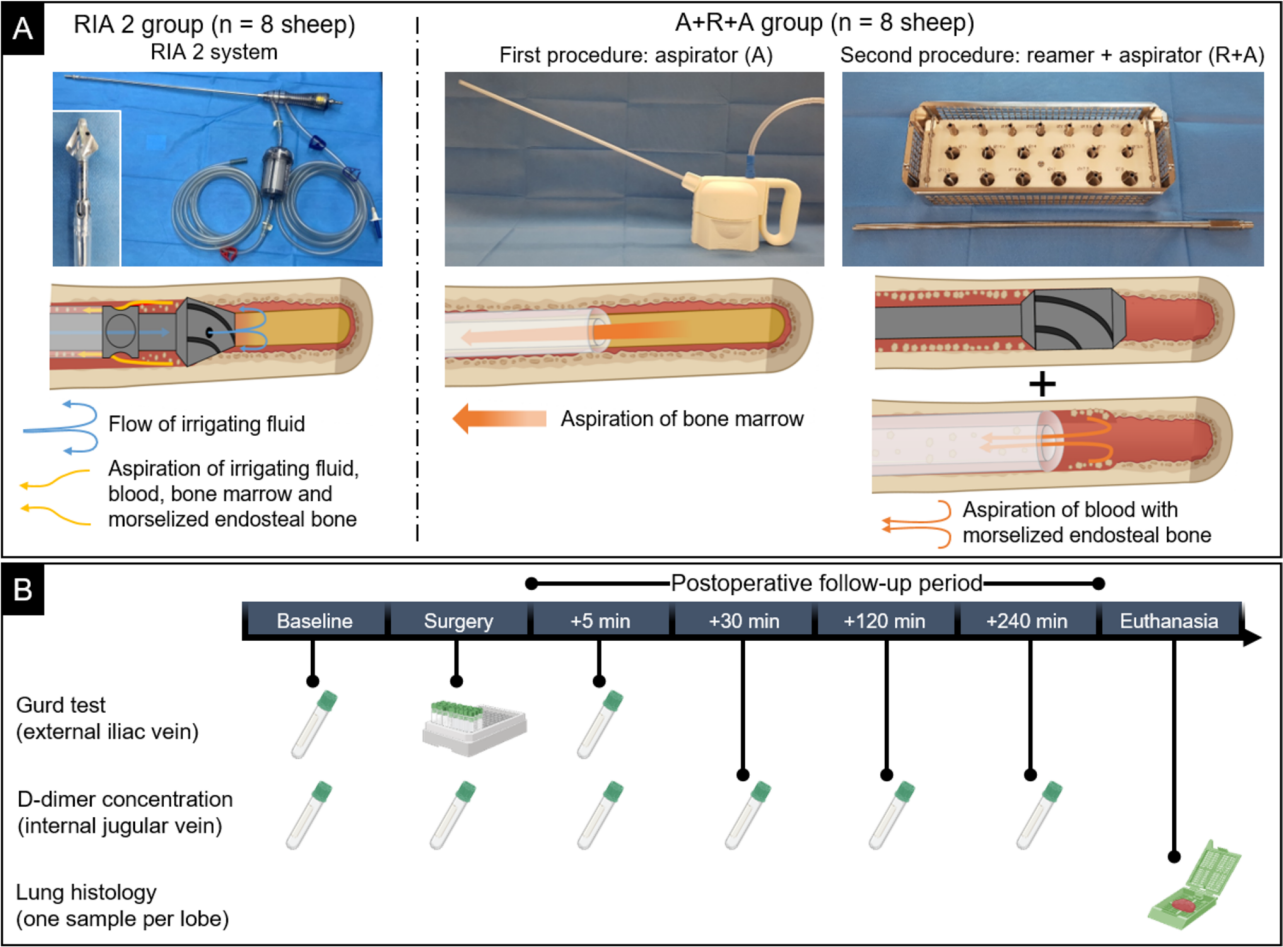
Experimental design and protocol. In the RIA 2 group, a one-step procedure with continuous flow of irrigation fluid during reaming, the RIA 2 system, is applied for BG harvesting, and in the A + R + A group, BM is first evacuated from the medullary canal using the aspirator followed by applying sequential IM reaming and aspiration of morselized endosteal bone chips using the aspirator (**A**). The experimental protocol illustrates the individual time points for the conducted assessments, including the Gurd test, D-dimer concentration, and lung histology (**B**). A is adapted from Ref. [3], and B is partially created with BioRender.com

In both experimental groups, BM fat intravasation was tested and quantified with the modified Gurd test during reaming procedures, coagulopathic response with D-dimer blood plasma concentration at multiple time points over 240 min after completion of surgery at defined intervals, and lung fat infiltration with histological analysis after euthanasia, which was performed using 100 mg/kg pentobarbital sodium (Lethabarb®) intravenously. Furthermore, postmortem, the sheep hearts were investigated for presence of atrial septal defects. Figure 1 depicts the experimental design and study protocol.

#### Gurd test for venous fat intravasation

Fat intravasation was measured by means of the Gurd test [28, 29], as modified by Schlag and Sommogy (1972) [30], in *n* = 6 sheep. At specific steps during the surgical procedure, blood samples were collected from the CVC positioned in the left EIV and the citrated blood samples centrifuged. Subsequently, the plasma was filtered through a 3-μm-pore cellulose nitrate filter and the filter plate stained with Nile Blue A, providing a dark background with fat globules appearing as bright yellow‒green disks when examined with epifluorescence microscopy. Once stained a histochemical inclusion medium was applied to the filter plate and epifluorescence microscopy is performed using a motorised upright Olympus BX63 microscope (Suppl. Figure 1). Individual images (*n* = 441–930) were taken per sample (stained filter plate) and were automatically stitched together to create one large image. The stitched images were imported into ImageJ software (version 1.53 k Java version 1.8.0_172), and the particle count and size as well as total area covered with lipid droplets were determined (Suppl. Figure 2). The number of intravasated BM fat particles, area of the slide covered with fat globules and corresponding average Gurd rating per sample (Table 1) were determined.

**Table 1:** Modified scoring system of the Gurd rating to assess fat intravasation [7; 59]

Rating	Average BM fat globule diameter per sample
Gurd 0	No visible fat globules
Gurd 0.5	1–4 μm
Gurd 1	5–10 μm
Gurd 2	11–20 μm
Gurd 3	21–50 μm
Gurd 4	51–100 μm
Gurd 5	> 100 μm

An average diameter of BM fat globules per sample resulted in a corresponding Gurd rating

#### Plasma D-dimer concentrations to assess the coagulopathic response to fat embolism

D-dimer is a coagulative factor measured in venous blood and an early marker of fat intravasation [31], as well as a marker for screening post-reaming pulmonary complications due to fat intravasation [20, 32]. A correlation between the degree of coagulopathic response and the severity of BM FE has been shown [32–34]. General anaesthesia was maintained for 240 min after the completion of surgery to measure the blood concentration levels of D-dimers to capture the variation and progress of the D-dimer concentration [20, 35]. Blood samples were collected from all sheep from the jugular CVCs using 5 ml S-Monovette® Citrate 3.2% tubes. The citrated blood samples were centrifuged at 3000 g for 15 min at 4 °C, and plasma was collected and frozen (– 80 °C) in low protein binding tubes (Thermo Fisher). The D-dimer concentration in the samples was quantified by applying the enzyme-linked immunosorbent assay (ELISA) method and using a sheep-specific ELISA kit (MyBioSource, Inc.; cat# MBS026385). Two ELISA kits were used in accordance with the manufacturer’s instructions, one for each experimental group, with standards and samples in duplicate. The absorbance of the ELISA plates was read at 450 nm, a standard curve was created, and a four-parameter logistic (sigmoidal, 4-PL) curve fit was used to determine the sample and standard concentrations (GraphPad Prism 9.3.0, CA, USA).

#### Lung histological evaluation for pulmonary fat embolism

After euthanasia, lung specimens were harvested from all sheep and immediately fixed in 4% paraformaldehyde (PFA). Since lung FE has been described as being equally distributed in all lobes [36] and with the highest concentration reported in the periphery [37, 38], one specimen of approximately 1.0 cm × 1.0 cm × 0.3 cm [39] per lung lobe was harvested at standardized anatomical locations in the lung periphery, resulting in six samples per sheep (Suppl. Figure 3). Lung tissue samples were postfixed with 4% osmium tetroxide (OsO_4_) aqueous solution (ProSciTech, cat# C011-1010) to stain the lipid content in black and allow for subsequent quantification of fat infiltration following a protocol developed by Abramowsky et al. (1981) [40] and optimized by Arregui et al. (2019) [41] (Suppl. Figure 4). For quantitative analysis, the scanned slides were imported into ImageJ software and the average of the three sections per lung lobe for particle count and size as well as total area covered with lipid droplets were determined (Suppl. Figure 5).

### Statistical analysis

Statistical analyses were performed using *R* programming software (version 4.0.2; R Foundation for Statistical Computing, Vienna, Austria) with RStudio version 1.3.1073 (RStudio Inc., Boston, MA). A significance level of *p* = 0.05 was chosen. Assessment of normal distribution of data was performed using the Shapiro‒Wilk test. Normally distributed data were tested using an independent-samples *t* test. For independent nonnormally distributed data, the Wilcoxon signed rank test with continuity correction was performed. A linear mixed model (LMM) with restricted maximum likelihood estimation (REML) was used with the time points or lung lobes as well as experimental groups as fixed effects and the sheep as random effects. For post-hoc test comparisons between the experimental groups per time point/lung lobe and within experimental groups across time points/lung lobes, the *R* package ‘emmeans’ was used with Tukey’s adjustment method for multiple comparisons. Data are presented as the mean with standard deviation ( ±) for normally distributed data and median with interquartile range (IQR) for nonnormally distributed data. The statistical methods of analysis for this study were selected in accordance with the Research Methods Group at QUT.

## Results

As per the isthmus diameter of the femoral shaft, with the objective of achieving matching of sheep in both groups with similar IM diameters (*p* = 0.68), the sheep were assigned to the RIA 2 group (*n* = 8) with an isthmus diameter of 12.21 ± 0.37 mm and the A + R + A group (*n* = 8) with an isthmus diameter of 12.29 ± 0.41 mm. In total, 73 reaming sequential steps were performed in the RIA 2 group (9.13 ± 0.84 per sheep), and 74 reaming sequential steps were performed in the A + R + A group (9.25 ± 0.71 per sheep, *p* = 0.75). All 16 sheep tolerated the (surgical) procedures well and survived the observational period. Atrial septal defects were not detected in the postmortem heart examination.

### Gurd test for venous fat intravasation

**Fig. 2:**
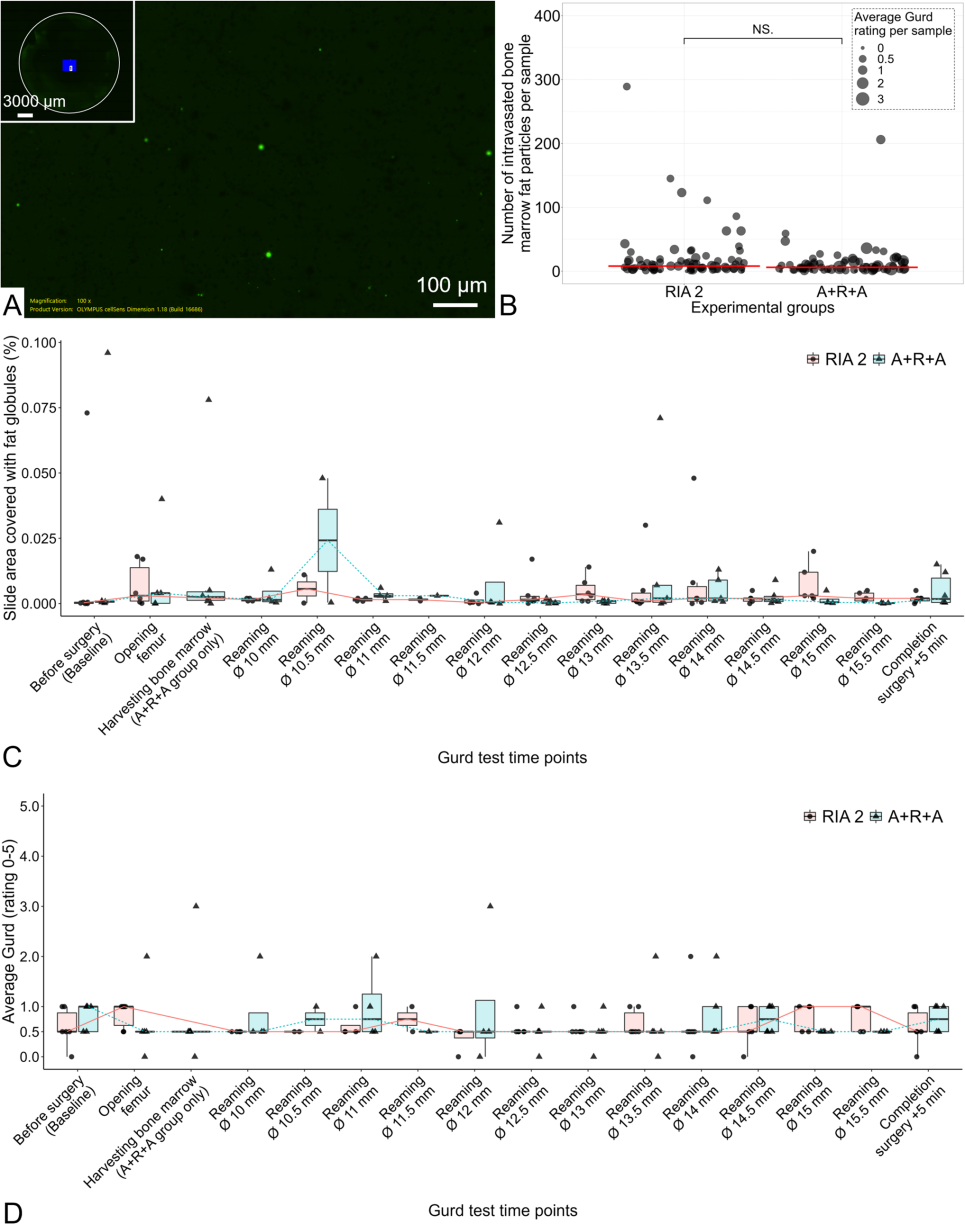
Evaluation of venous fat intravasation assessed with the modified Gurd test. Epifluorescence microscope image (magnification 100x), with a blue rectangle in the inset depicting the scanned area of the filter membrane, showing visible individual fat globules in an exemplary section of a sample that consists of a total of 289 particles ranging in diameter from 1.14 to 18.78 μm, resulting in a slide area covered with fat of 0.048% with a corresponding average median Gurd value of 0.5 (**A**). Overall, in the RIA 2 (*n* = 75) and A + R + A (*n* = 81) samples, no differences in the median number of bone marrow fat particles per sample (*p* = 0.13) and Gurd rating per sample (*p* = 0.98) were observed (B, red crossbar = median). The effect seen in the experimental groups on the area of the microscope slide covered with fat globules (**C**) was not different either for the comparison between the experimental groups per time point or within the respective experimental group in the comparison of the different time points (all *p* > 0.05). Moreover, there was no difference in the Gurd rating (**D**) between the two groups at the respective time point or within the groups between the time points (all *p* > 0.05). *NS,* nonsignificant

The total number of intravasated BM fat particles per sample was not significantly different (*p* = 0.13) between the RIA 2 (8, IQR 14) and A + R + A group (6, IQR 11.8). Moreover, the overall average Gurd rating per sample was not found to be significantly different (*p* = 0.98) between the RIA 2 (0.5, IQR 0.5) and A + R + A group (0.5, IQR 0.5). The median Gurd rating of 0.5 with an IQR of 0.5 in both groups indicates that overall small fat particles were intravasated perioperatively. Furthermore, no differences per time point between the RIA 2 and A + R + A groups or within the experimental groups were found when comparing the different time points and concerning fat intravasation illustrated as the area of microscope slide covered with fat globules and the fat particle diameter as evaluated with Gurd rating (all *p* > 0.05, Suppl. Tables 1 and 2). Thus, venous fat intravasation in terms of particle number and size was not different between the groups and were similar when comparing them within the different interventional stages (Fig. 2). For the first reaming step alone, there was also no significant difference in the number of FE particles or in the Gurd rating between the two groups (*p* = 1 and *p* = 0.18, respectively) (Suppl. Figure 6).

### Plasma D-dimer concentrations to assess the coagulopathic response to fat embolism

**Fig. 3:**
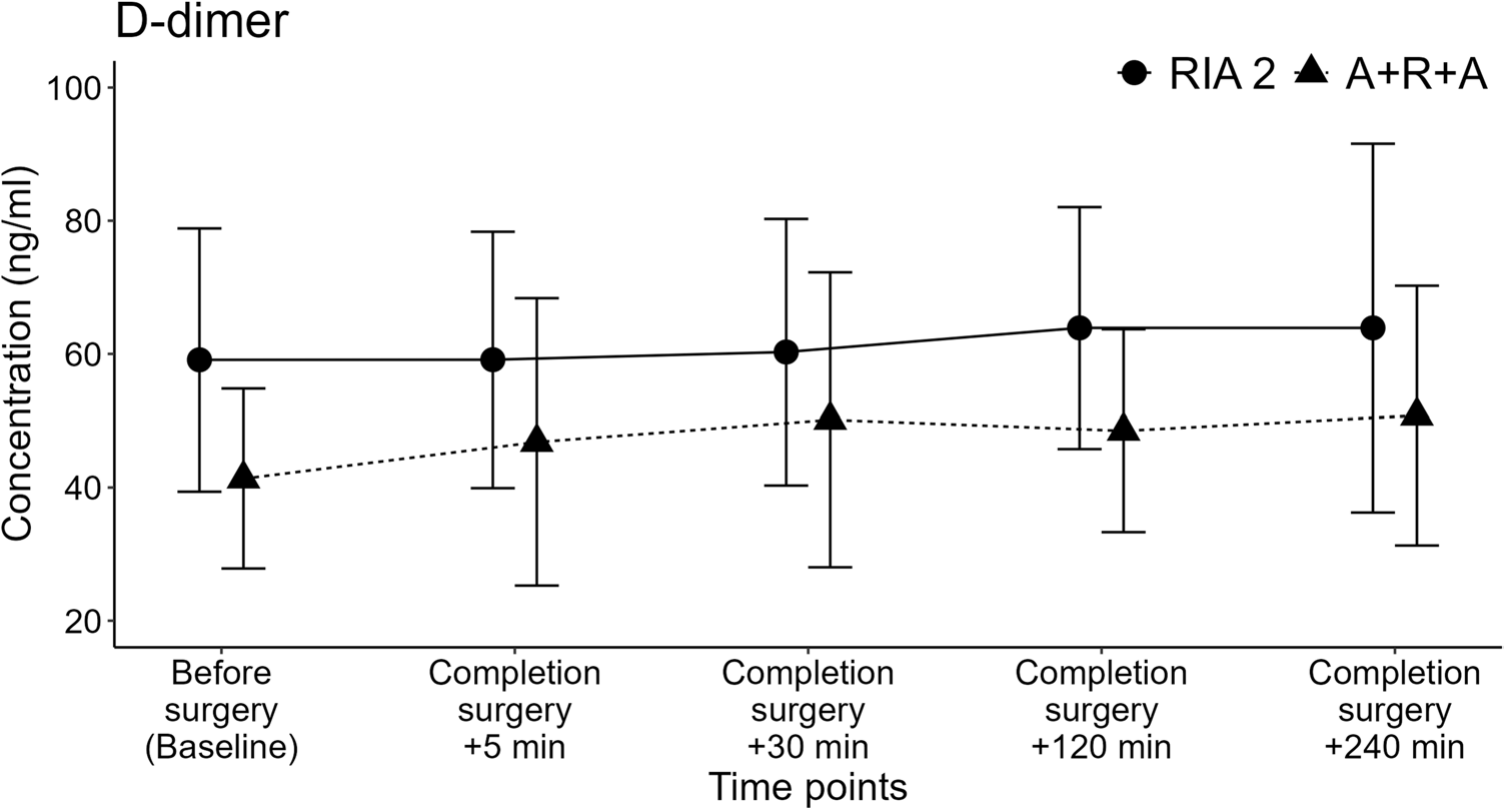
D-dimer concentrations measured in the blood plasma samples obtained from the experimental groups at multiple time points for up to 4 h after completion of surgery. No significant different interaction effect was observed on D-dimer concentration over time when comparing the RIA 2 with the A + R + A group (*p* = 0.65). The data are displayed as mean ± SD

D-dimer concentration increased 8.1% from baseline in the RIA 2 group, reaching 63.90 ng/ml (mean difference 4.80 ng/ml) from 59.11 ng/ml 240 min after completion of surgery (*p* = 0.82), and increased 22.8% from baseline in the A + R + A group, reaching 50.77 ng/ml (mean difference 9.44 ng/ml) from 41.33 ng/ml 240 min after completion of surgery (*p* = 0.23). The baseline differences are related to the fact that ELISA assays were performed at different assay dates for each group. No significant difference was observed across all time points when comparing the interaction effects of the respective experimental groups (*p* = 0.65) on D-dimer concentration (Fig. 3). Moreover, the post-hoc test showed that there were no significant differences in D-dimer concentration between the different time points within each experimental group (all *p* > 0.05, Suppl. Table 3).

#### Lung histological evaluation for fat embolism

**Fig. 4:**
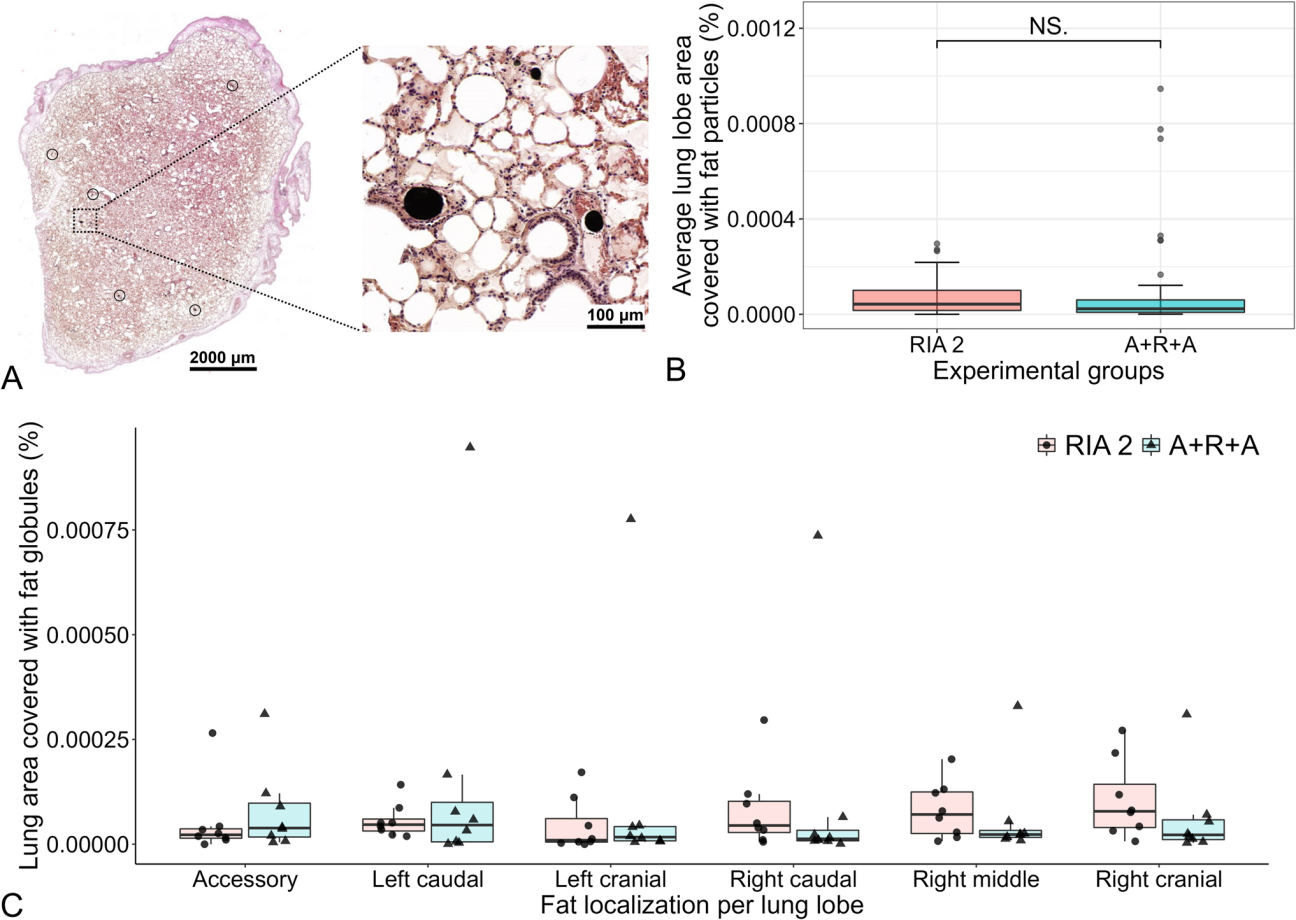
Histological results for sheep lung lobe area covered with fat globules. During fixation with lipids, OsO_4_ is reduced to lower oxides that are black and insoluble and are deposited in tissues. The intravascular lipids appeared as single or multiple, well-defined and black (OsO_4_) globules (**A**). The illustrated section in A includes the lung lobe area covered on this section, which was 0.006% of the total stained area with an average particle size of 878.003 μm^2^ and in total eight particles (indicated by black circles) identified as fat globules. No difference between the RIA 2 and A + R + A groups (*n* = 48) for average lung lobe area covered with fat globules (*p* = 0.17) was observed (**B**). For the area covered with fat, no significant differences (all *p* > 0.05) were observed for the individual lung lobes either within a group or between the experimental groups (**C**). *NS*, nonsignificant. The data in panels B and C are displayed as boxplots with individual data points superimposed in panel C

The average lung area for all lung lobes covered with fat when comparing the RIA 2 group (0.0000425%; IQR 0.0000842) and A + R + A group (0.0000229%; IQR 0.0000518) was not significantly different (*p* = 0.17). Furthermore, no significant differences were observed for lung area covered with fat when comparing the different lung lobes within and between groups (all *p* > 0.05, Suppl. Table 4) (Fig. 4). Moreover, no difference in average particle size (*p* = 0.34) and number (*p* = 0.14) was observed when comparing both groups (Suppl. Figure 7). The detailed analysis of the individual lung lobes also revealed no significant differences between the lung lobes in comparison within as well as between the experimental groups with regard to size and number of fat particles (all *p* > 0.05, Suppl. Tables 5 and 6) (Suppl. Figure 8). Thus, BM FEs found lodged in the sheep lungs were not different between experimental groups and equally distributed between lobes.

## Discussion

Since graft materials retrieved from the medullary canal of long bones are associated with high bone regenerative capacity, academia and industry are advancing harvesting concepts [42–44]. Laubach et al. (2023) [3] recently reported an innovative approach for harvesting IM BG, using a prototype of an aspirator that allows staged harvesting of BM and morselized endosteal bone from the IM canal of long bones. In the current work, we tested this novel concept (A + R + A group) in a nonfracture sheep femur model to assess the complication of BM fat intravasation and observed no differences compared to the RIA 2 system (RIA 2 group). Notably, the RIA system was first developed to reduce the incidence of (cortical and systemic) FE during reamed femoral facture nailing [21, 45] and only later was approved by the FDA in 2005 for obtaining autologous graft material due to its large BG harvesting capacity [46]. In both the RIA 2 and A + R + A groups, only minor venous fat intravasation, coagulopathic response and lung FE were observed, supporting our hypothesis that the novel alternative harvesting concept results in comparable (minor) levels of BM FE to the RIA 2 system.

Volume displacement is inevitable in a confined space (e.g., IM cavity) in accordance with Pascal’s law. The amount of embolism has been shown to be directly correlated with the IM pressure level [10]. The highest increase in IM pressure is observed during the first reaming steps [47]. Important variables to overcome the effects of Pascal’s law were published by Stürmer (1993) [48] (“gap equation” formula) to illustrate potential factors that may be addressed to decrease pressure while operating in the IM canal (Suppl. Figure 9). In the gap equation formula, the viscosity of the IM content is associated with a factor of 12, which means that a significant IM pressure reduction is achieved as the fluid viscosity decreases. The theoretical underlying working principles of the RIA system to reduce FE are based on a number of parameters mainly associated with IM pressure reduction, particularly decreased BM viscosity [21]. However, in a clinical study, no advantage of the RIA system over standard reaming for femur fracture regarding complications such as cortical BM FE and delayed union or nonunion was observed [49, 50]. Therefore, the question arises whether extracting BM before reaming is preferable because it more effectively reduces IM pressure, while reducing BM viscosity during RIA-reaming is not sufficient to reduce IM pressure. In particular, the RIA system generates higher pressures in the distal metaphysis than in the middle diaphysis of the tibia, which contradicts a generalization of the RIA system IM pressure reduction theory [51].

The Gurd test was performed with a venous cannula inserted into the sheep left EIV aiming at lesser FE dilution compared to the use of the central vein. Post-surgery, the sheep remained anaesthetized for 240 min for blood analyses, including the early coagulopathy marker for fat intravasation, D-dimer. [31]. Earlier literature suggests monitoring D-dimer levels for 4 h post-IM reaming [20]. We observed very low Gurd test ratings, consistent with results from reaming systems such as the Bixcut reamer [7] and the RIA system [52]. Applying the RIA system prior to femoral medullary nailing in sheep with unilateral pulmonary injury reduced D-dimer concentrations compared to standard reaming [20]. These findings align with the minimal FE and stable D-dimer concentration observed in our study, indicating no significant differences between the groups. Based on the present study, the new harvesting concept A + R + A is comparable to the RIA 2 system in terms of fat intravasation and coagulopathic response.

Several large animal studies on lung embolism have been performed, each with different evaluation methods, making direct comparisons a challenge [39, 52, 53]. In our study, we standardized the retrieval of the anatomical samples and used an average of three sections per lung lobe for detailed fat particle analysis. Our semiautomated image analysis protocol (Suppl. Figure 5) ensured highly reproducible results. The 240 min perioperative follow-up aligns with previous research showing the greatest FE concentration within the first day post-surgery [37, 54], with disappearance from the alveolar capillaries within 72 h [37, 55]. This time period allowed our sheep model to effectively capture the majority of the BM lung FE, indicating a low total lung emboli load in both experimental groups. Previous studies have shown reduced lung FE with the RIA system [52, 56] and BM removal [24] compared to reaming without BM evacuation. Our study is the first to show that the combination of BM removal followed by standard reaming (A + R + A) results in similarly low FE rates as the RIA 2 system.

IM reaming systems underwent many modifications during the last 25 years, including smaller cored diameter reamer heads with increased clearance area, such as the Bixcut IM Reamer System (Stryker, Kalamazoo, MI), with reduced IM reaming pressure compared to other types of standard reamers [47, 57, 58]. While previous studies with a standard AO/ASIF reamer (Synthes, Germany) observed relevant FE [52, 59] in comparison with the RIA system [20, 56], recent studies have shown that deeper flute designs exert lower pressure because they allow for easier escape of the debris/contents from cutting [7, 39, 53]. Additionally, in comparison with the RIA 2 system, the Bixcut reamer has a deeper reamer head with large forwards and side cutting flutes and a thinner shaft (third power in the numerator in the “gap formula”), which in turn could result in higher throughput of medullary content and thus lower IM pressure according to Pascal’s law. Therefore, our results also provide an incentive to investigate, in further studies, the extent to which the combination of BM removal and advanced reamer head design (e.g., Bixcut reamer) can contribute to the problem of cortical (fat acts as a mechanical block and as a toxic factor preventing revascularization [60]) and systematic fat intravasation in reamed nailing fractures leading to delayed union or nonunion, which has already been discussed in great detail by Danckwardt and colleagues for more than 50 years [60] and has not been solved by applying the RIA system for reamed nailing [49, 61].

### Limitations

The sheep femur model used in this study is not fully comparable to the results obtained in the human femur. Keeping with Pascal’s law (“gap equation”), which predicts that a piston acting in a longer cylinder will create higher pressure than in a shorter cylinder, the greater the length of intact bone is, the higher the pressure [17]. A sheep femur is shorter than a human femur (14.5 cm vs. 42.6 cm) [62], and its IM contents represent only approximately one-third of the volume of the IM contents of the human femur in relation to body size [20]. Therefore, comparing BM FE with those seen in humans is limited, even in a nonfracture model that prevents escape of BM from the fracture gap. We initially considered using sheep tibias. Sheep tibia is narrower than the femur and therefore could have resembled a more challenging model resulting in more BM FE. However, even the smallest reamer head size of the RIA 2 system (10 mm in diameter) was not suitable in this animal model [3]. Furthermore, we observed similar fat intravasation across all reaming steps and between experimental groups, which might be associated with a rather conservative graft material harvesting approach by starting reaming 2 mm narrower than the isthmus, which was chosen to allow the safest possible assessment of the novel harvesting concept. However, in clinical practice, the commencement of harvesting with a reamer head size equal to that of the isthmus might be preferred, especially as this reduces the number of reamer heads used and thus costs.

## Conclusion

In a sheep femur model, the novel harvesting concept using an innovative aspirator prototype for BM evacuation followed by harvesting of endosteal morselized bone with sequential reaming steps resulted in similar low amounts of BM fat intravasation, coagulopathic response and lung FE compared to the use of the RIA 2 system and thus represents a safe alternative for IM BG harvesting.

### Supplementary Information

Below is the link to the electronic supplementary material.

Supplementary file1 (DOCX 4914 KB)

## Data Availability

The data that support the findings of this study are available from the corresponding author upon reasonable request.
