# Supplementary material for: In vivo study to assess fat embolism resulting from the Reamer-Irrigator-Aspirator 2 system compared to a novel aspirator-based concept for intramedullary bone graft harvesting
Source: Arch Orthop Trauma Surg. 2024 Feb 17;144(4):1535–46. doi: 10.1007/s00402-024-05220-w (PMC10965743; doi:10.1007/s00402-024-05220-w)
Supplement: Supplementary file 1 — Supplementary file1 (DOCX 4914 KB) [file 402_2024_5220_MOESM1_ESM.docx]

Supplement

Contents

[Supplementary figures 2](#_Toc154220330)

[Suppl. Fig. 1. Gurd test setup and procedure. 2](#_Toc154220331)

[Suppl. Fig. 2. ImageJ workflow for evaluating Gurd test images with intravasated bone marrow fat globules. 4](#_Toc154220332)

[Suppl. Fig. 3. Example cadaver illustration of a sheep lung with distinguishable lobes and designated sampling sites. 5](#_Toc154220333)

[Suppl. Fig. 4. Applied protocol for fixation, postfixation staining (steps 1-7, light green), block tissue processing and slide scanning (steps 8-11, dark green) of lung tissue samples. 6](#_Toc154220334)

[Suppl. Fig. 5. ImageJ workflow for quantitative analyses of lipid globules in scanned lung histology slides. 7](#_Toc154220335)

[Suppl. Fig. 6. Evaluation of venous fat intravasation for the first reaming step assessed by number of intravasated BM fat particles and by means with modified Gurd test. - 8 -](#_Toc154220336)

[Suppl. Fig. 7. Boxplots of average particle size and numbers to assess pulmonary fat embolism. - 9 -](#_Toc154220337)

[Suppl. Fig. 8. Boxplots of average fat particle size and number plotted for the individual lung lobes. - 10 -](#_Toc154220338)

[Suppl. Fig. 9. Gap equation to illustrate effects of Pascal’s Law as published by Stürmer [5]. - 11 -](#_Toc154220339)

[Supplementary tables - 12 -](#_Toc154220340)

[Suppl. Table 1. Area of slide covered with fat globules compared between experimental groups per time point and within groups over different time points. - 12 -](#_Toc154220341)

[Suppl. Table 2. Average Gurd rating compared between experimental groups per time point and within group over different time points. - 18 -](#_Toc154220342)

[Suppl. Table 3. Differences in D-dimer concentration between the different time points within each experimental group. - 23 -](#_Toc154220343)

[Suppl. Table. 4. Percentage area covered with fat of each lung lobe between experimental groups or within a group. - 24 -](#_Toc154220344)

[Suppl. Table 5. Average size of fat particles in individual lung lobes either between experimental groups or within a group. - 26 -](#_Toc154220345)

[Suppl. Table 6. Average number of fat particles in individual lung lobes either between experimental groups or within a group. - 27 -](#_Toc154220346)

[References - 29 -](#_Toc154220347)

# Supplementary figures

## Suppl. Fig. 1. Gurd test setup and procedure.


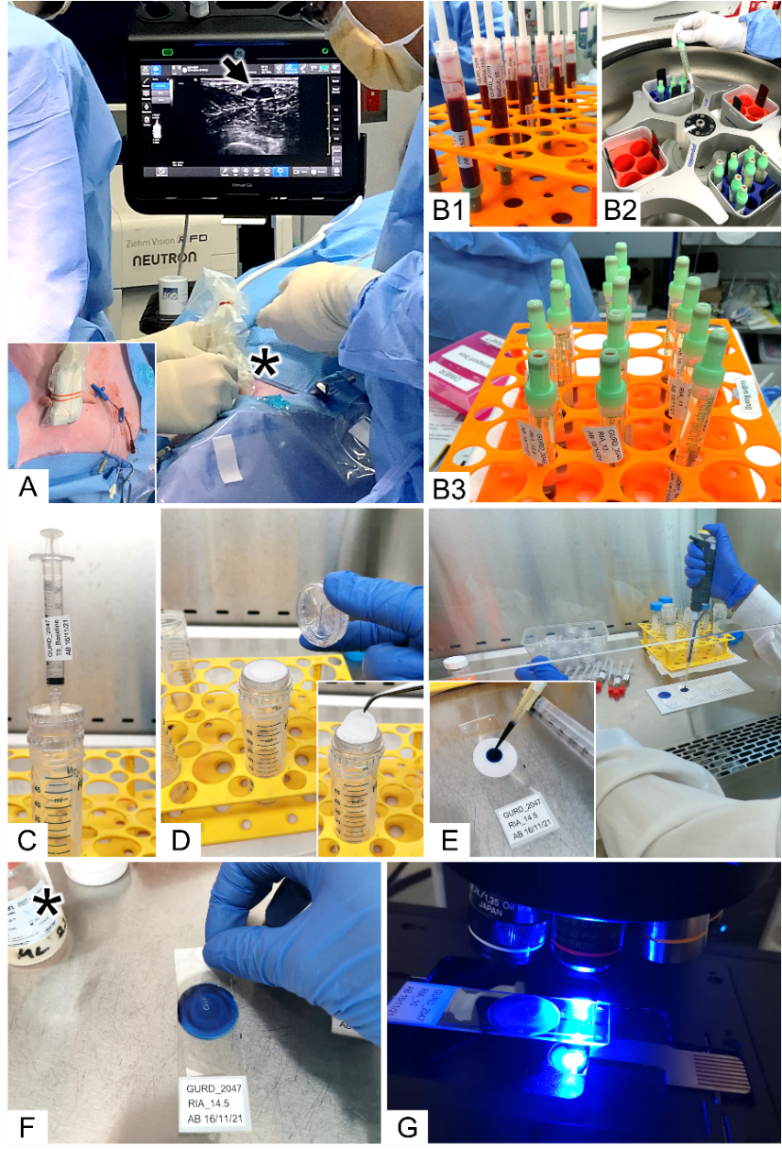


A: A central venous catheter (CVC, 8.5 Fr.) is placed in left external iliac vein (EIV) using Seldinger technique. Black arrow in A points to left EIV where diameter is slightly reduced by inserted introducer needle, and the asterisk (*) is indicating the spring-wire guide with arrow advancer. Inset in A shows CVC *in situ* before sutured to the skin. Following insertion of the CVC in the EIV the sheep were re-positioned in right lateral recumbency, and the skin was aseptically prepared in preparation for surgery, as previously described [1; 4] while ensuring sufficient accessibility to the hip joint for the anterograde approach of the left proximal femur. B1-3: Blood samples (B1) taken from the left EIV using 5 ml S-Monovette® Citrate 3.2% tubes (Sarstedt, cat# 05.1071) during the surgical interventions are centrifuged (B2) at 3000g for 15 min at 21 °C and 2 ml of plasma (B3) aspirated into a fat-free disposable syringe using a large bore (16 g) needle (ZebraVet Australia Pty Ltd, cat# BDN16). C: The plasma is filtered through a 3-μm-pore cellulose nitrate filter with a diameter of 25 mm (Sartorius, cat# 11302-25) mounted in a polycarbonate syringe filter holder (Sartorius, cat# 16517). D: The filter membrane transferred (inset in D) to a microscope slide. E: The filter plate was then transferred to a microscope slide and stained with a 1% aqueous solution of Nile Blue A (75 µl, Sigma‒Aldrich, cat# N0766-25G) using a pipette and sterilized tips for standardized staining volume (inset in E). F: Once stained, 100 µl Fluoromount-G™ (Thermo Fisher, cat# 00-4958-02) was used to mount slides (* in F), kept in the dark and imaged with an epifluorescence microscope within a short time. G: An epifluorescence microscope (motorized upright Olympus BX63 microscope) with a standard FITC filter set for imaging (excitation filter 450 nm, emission filter 520 nm, 100× objective lens, exposure time 60 ms, gain 16×) was used for imaging the fat trapped on the filter plate.

## Suppl. Fig. 2. ImageJ workflow for evaluating Gurd test images with intravasated bone marrow fat globules.


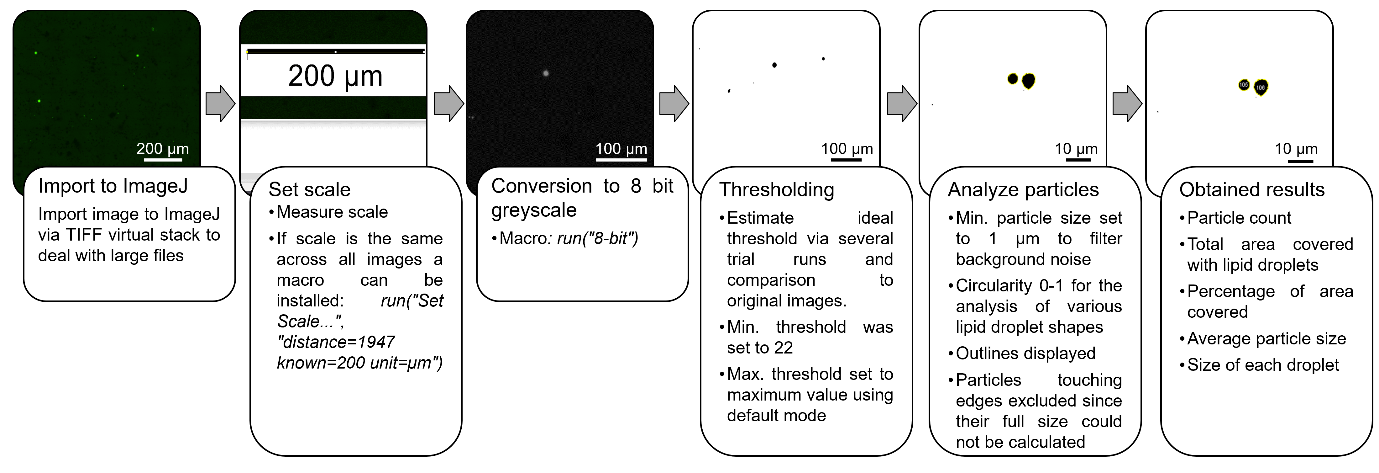


Image files obtained with the Olympus BX63 microscope are imported in ImageJ via TIFF virtual stack to deal with large image sizes. The scale was the same across all images, therefore, a macro was installed to set the scale for each image and to convert the image to greyscale (8-bit) to allow thresholding. The ideal threshold was estimated via several trial runs and comparison to the original images. In accordance with the literature the threshold minimum set to 22 [2] and maximum set to the maximum image value, using the default mode (default mode = IsoData algorithm, iterative intermeans, iterative procedure based on the isodata algorithm). The minimum particle size was set to 1 μm to filter background noise, circularity 0-1 allowed for the analysis of various shapes of lipid droplets. Subsequently, outlines of the analyzed particles were displayed and particles touching the edges were excluded since their full size could not be calculated. Results were exported as .csv file. Furthermore, data of analyzed particles from the region of interest (ROI) manager was added to the image file and saved as TIFF file.

## Suppl. Fig. 3. Example cadaver illustration of a sheep lung with distinguishable lobes and designated sampling sites.


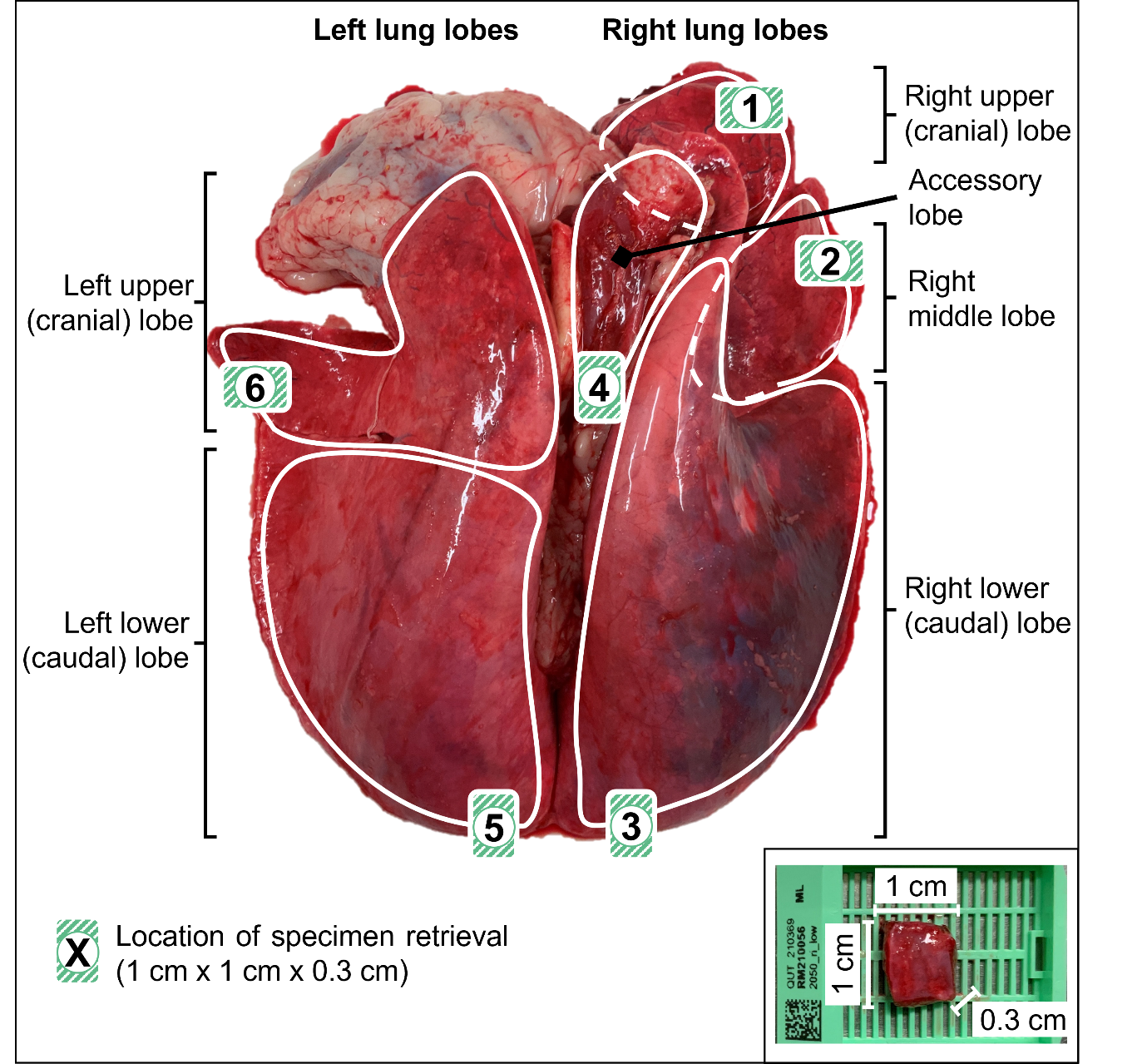


The left and right sides of the sheep lung comprise six distinct lobes separated by tissue septa. The right lung contains four lobes, while the left lung contains two lobes [3]. Standardized for anatomical localization and sample size one specimen was taken from each of the six lung lobes. An exemplary picture of a specimen retrieved directly after humane killing is shown in the inset.

## Suppl. Fig. 4. Applied protocol for fixation, postfixation staining (steps 1-7, light green), block tissue processing and slide scanning (steps 8-11, dark green) of lung tissue samples.

## Suppl. Fig. 5. ImageJ workflow for quantitative analyses of lipid globules in scanned lung histology slides.


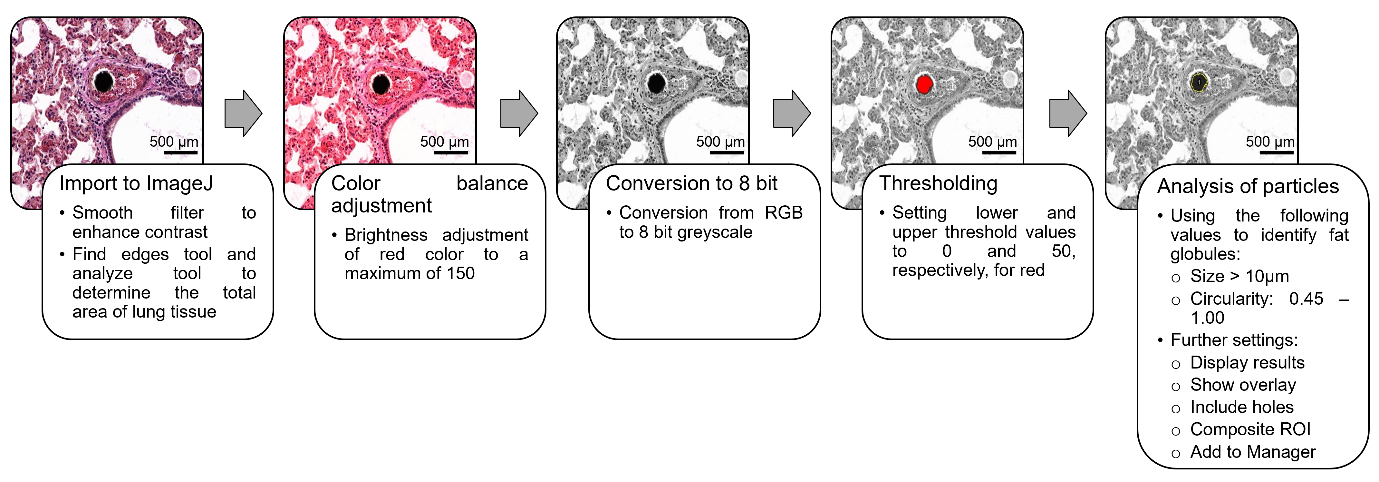


Microscope slides were retrieved from the SlideViewer software (3DHISTECH Ltd., Slide Converter 2.2) by exporting snapshots with slide information and scale. In ImageJ the image was imported using ImageScope for further analyses as illustrated.

## Suppl. Fig. 6. Evaluation of venous fat intravasation for the first reaming step assessed by number of intravasated BM fat particles and by means with modified Gurd test.


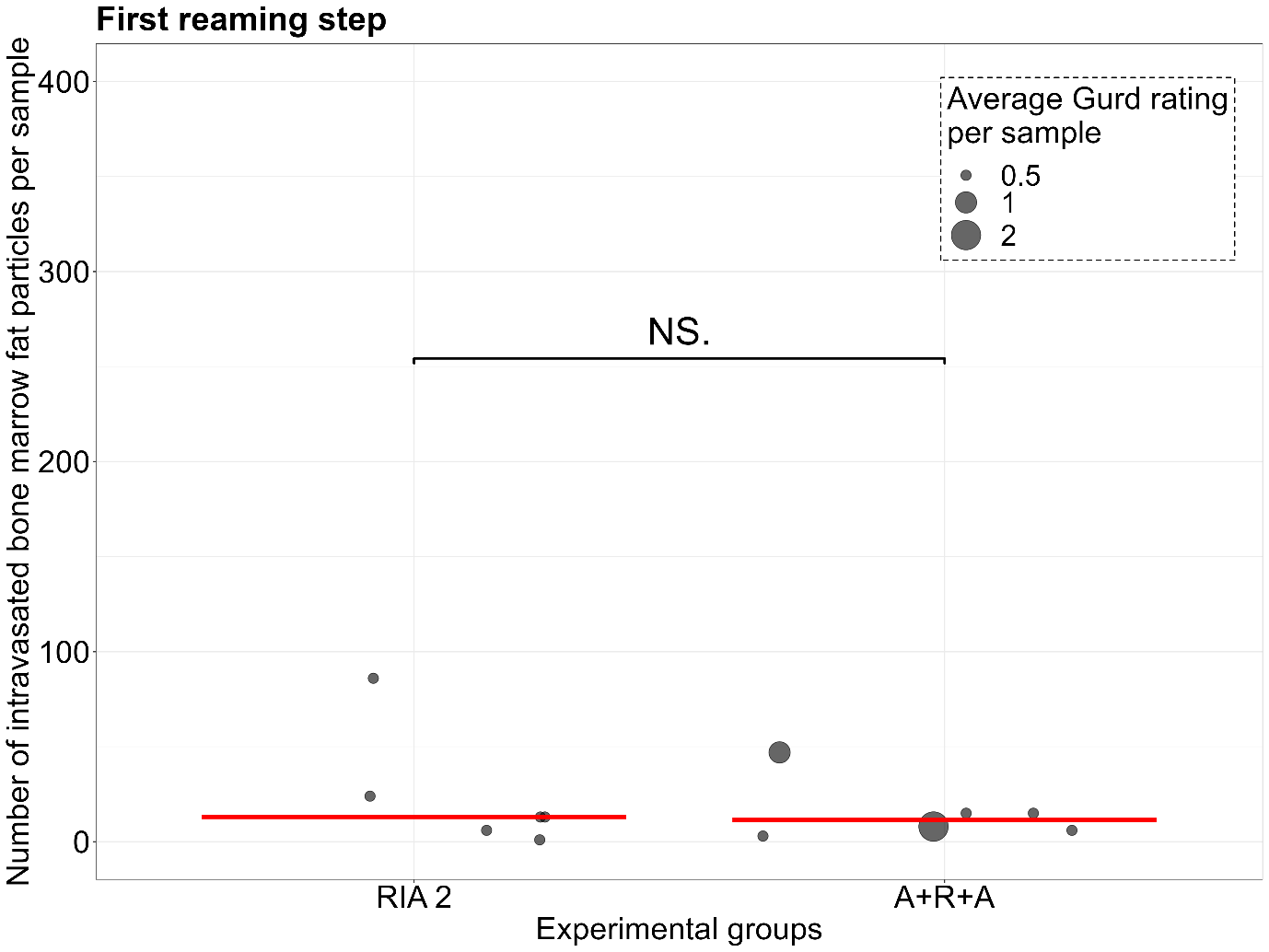


Samples were assessed for intravasated bone marrow fat particles using the modified Gurd test (n = 6). No differences in median number of bone marrow fat particles per sample in RIA 2 group of 13 (IQR 0.35) and in A+R+A group of 11.50 (IQR 18, *p* = 1) and Gurd rating per sample in RIA 2 group of 0.5 (IQR 0.0) and in A+R+A group 0.5 (IQR 0.8, *p* = 0.18) were observed (red crossbar = median). BM, bone marrow. NS. = nonsignificant.

## Suppl. Fig. 7. Boxplots of average particle size and numbers to assess pulmonary fat embolism.


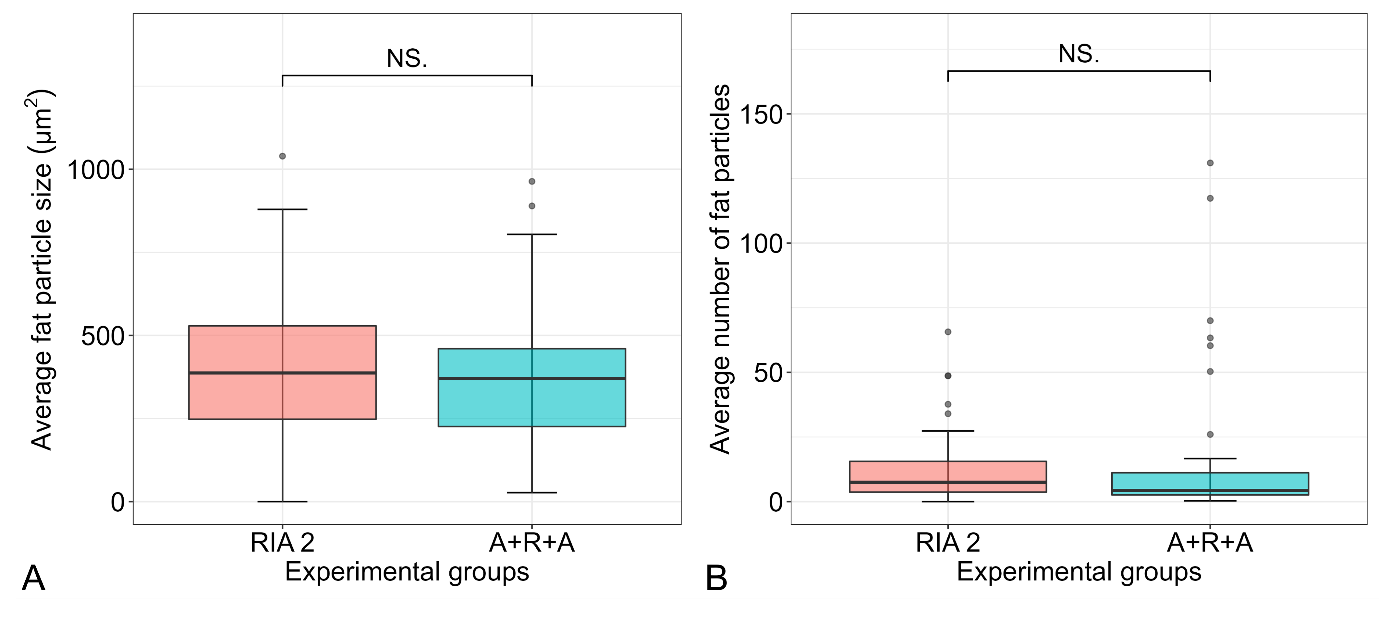


A: No significant difference (*p* = 0.34) was observed for the average fat particle size n = 48 per group) comparing RIA 2 group (387.16 μm^2^; IQR 280.87) with A+R+A group (370.61 μm^2^; IQR 234.02). B: Also, there was no significant difference (*p* = 0.14) for the average number of fat particles (n = 48 per group) between groups (RIA 2 group: 7.50 μm^2^; IQR 11.92 and A+R+A group: 4.34 μm^2^; IQR 8.58). NS. = nonsignificant.

## Suppl. Fig. 8. Boxplots of average fat particle size and number plotted for the individual lung lobes.


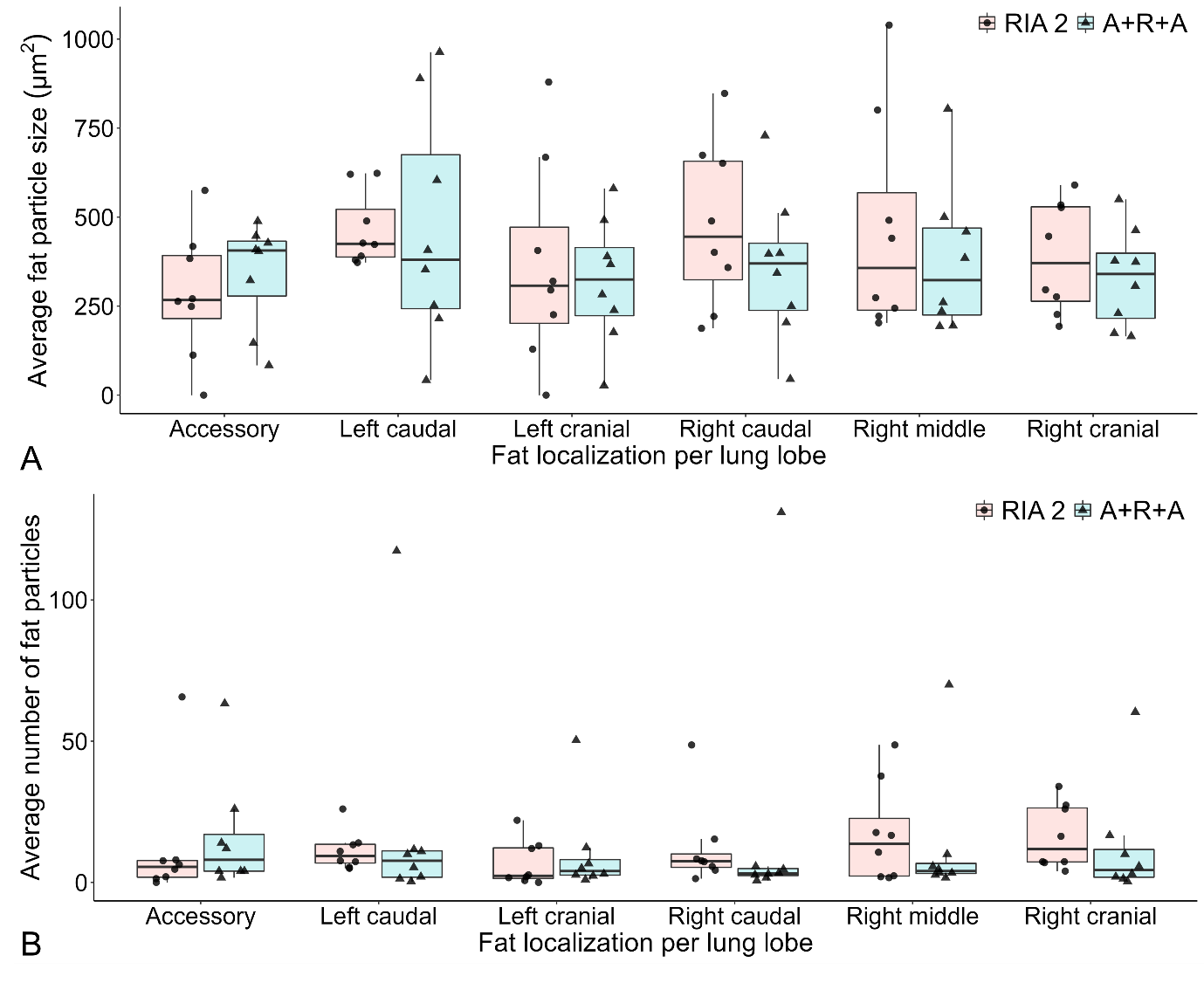


Statistical analysis using the LMM with post-hoc test (Tukey method correction) revealed no difference in fat particle size (A) and number (B) for the individual lung lobes either within or between the experimental groups (all *p* > 0.05). The data are displayed as boxplots with individual data points superimposed.

## Suppl. Fig. 9. Gap equation to illustrate effects of Pascal’s Law as published by Stürmer [5].


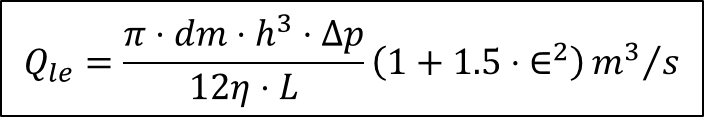


$Q_{le}$ = flow rate; dm = average diameter of the bore and piston; h = gap [cm]; $\Delta p$ = pressure difference [N/m^2^]; L = length of seal [cm]; $\eta$ dynamic viscosity [Ns/m^2^]; $\in$ = $\frac{e}{h}$ relative eccentricity; and e = eccentricity [cm].

Overall, the flow out of the chamber must be equal to or greater than that generated in the confined space to prevent positive pressure from being created. The flow rate (Q_le_) in the “gap equation” of this hydraulic system is the key to achieving IM space manipulation without a significant pressure increase [6].

# Supplementary tables

## Suppl. Table 1. Area of slide covered with fat globules compared between experimental groups per time point and within groups over different time points.

|  | **RIA 2 group** | | | **A+R+A group** | | **Comparison experimental (RIA 2/A+R+A) groups** | |
| --- | --- | --- | --- | --- | --- | --- | --- |
| **Time point** | **Estimated mean (standard error)** | **95% Confidence interval (lower / upper)** | | **Estimated mean (standard error)** | **95% confidence interval (lower / upper)** | **Estimated mean difference (standard error** | **P - value** |
| Before surgery (Baseline) | 0.012367 (0.00637) | (-0.000263 / 0.0250) | | 0.020006 (0.00698) | (0.006174 / 0.0338) | -0.007639 (0.00945) | 0.4206 |
| Opening femur | 0.006950 (0.00637) | (-0.005680 / 0.0196) | | 0.009946 (0.00698) | (-0.003886 / 0.0238) | -0.002996 (0.00945) | 0.7518 |
| Harvesting bone marrow | NA | NA | | 0.014833 (0.00637) | (0.002204 / 0.0275) | NA | NA |
| Reaming ⌀ 10 mm | 0.001322 (0.00779) | (-0.014114 / 0.0168) | | 0.004268 (0.00779) | (-0.011177 / 0.0197) | -0.002946 (0.01102) | 0.7897 |
| Reaming ⌀ 10.5 mm | 0.005956 (0.01100) | (-0.015835 / 0.0277) | | 0.023765 (0.01101) | (0.001952 / 0.0456) | -0.017809 (0.01556) | 0.2549 |
| Reaming ⌀ 11 mm | 0.001297 (0.00779) | (-0.014139 / 0.0167) | | 0.003468 (0.00779) | (-0.011977 / 0.0189) | -0.002171 (0.01102) | 0.8442 |
| Reaming ⌀ 11.5 mm | 0.001856 (0.01100) | (-0.019935 / 0.0236) | | 0.003759 (0.01551) | (-0.026984 / 0.0345) | -0.001903 (0.01902) | 0.9205 |
| Reaming ⌀ 12 mm | 0.001022 (0.00779) | (-0.014414 / 0.0165) | | 0.008218 (0.00779) | (-0.007227 / 0.0237) | -0.007196 (0.01102) | 0.5151 |
| Reaming ⌀ 12.5 mm | 0.004017 (0.00637) | (-0.008613 / 0.0166) | | 0.000986 (0.00698) | (-0.012846 / 0.0148) | 0.003031 (0.00945) | 0.7490 |
| Reaming ⌀ 13 mm | 0.005150 (0.00637) | (-0.007480 / 0.0178) | | 0.000051 (0.00697) | (-0.013769 / 0.0139) | 0.005099 (0.00944) | 0.5904 |
| Reaming ⌀ 13.5 mm | 0.006250 (0.00637) | (-0.006380 / 0.0189) | | 0.016426 (0.00698) | (0.002594 / 0.0303) | -0.010176 (0.00945) | 0.2839 |
| Reaming ⌀ 14 mm | 0.010083 (0.00637) | (-0.002546 / 0.0227) | | 0.004571 (0.00697) | (-0.009249 / 0.0184) | 0.005512 (0.00944) | 0.5607 |
| Reaming ⌀ 14.5 mm | 0.002989 (0.00697) | (-0.010826 / 0.0168) | | 0.002650 (0.00637) | (-0.009980 / 0.0153) | 0.000339 (0.00944) | 0.9715 |
| Reaming ⌀ 15 mm | 0.007949 (0.00697) | (-0.005869 / 0.0218) | | 0.001246 (0.00779) | (-0.014195 / 0.0167) | 0.006703 (0.01046) | 0.5228 |
| Reaming ⌀ 15.5 mm | 0.002227 (0.00697) | (-0.011587 / 0.0160) | | -0.001030 (0.00898) | (-0.018825 / 0.0168) | 0.003257 (0.01137) | 0.7750 |
| Completion surgery +5 min | 0.001950 (0.00637) | (-0.010680 / 0.0146) | | 0.005217 (0.00637) | (-0.007413 / 0.0178) | -0.003267 (0.00901) | 0.7176 |
| **RIA 2 group** | | | | | | | |
| **Time points** | | | | | **Estimated mean difference** | **Standard error** | **P - value** |
| Before surgery (Baseline) | | | Opening femur | | 0.005417 | 0.00868 | 1.0000 |
| Before surgery (Baseline) | | | Harvesting bone marrow | | NA | NA | NA |
| Before surgery (Baseline) | | | Reaming ⌀ 10 mm | | 0.011045 | 0.00977 | 0.9989 |
| Before surgery (Baseline) | | | Reaming ⌀ 10.5 mm | | 0.006411 | 0.01248 | 1.0000 |
| Before surgery (Baseline) | | | Reaming ⌀ 11 mm | | 0.01107 | 0.00977 | 0.9989 |
| Before surgery (Baseline) | | | Reaming ⌀ 11.5 mm | | 0.010511 | 0.01248 | 1.0000 |
| Before surgery (Baseline) | | | Reaming ⌀ 12 mm | | 0.011345 | 0.00977 | 0.9986 |
| Before surgery (Baseline) | | | Reaming ⌀ 12.5 mm | | 0.00835 | 0.00868 | 0.9998 |
| Before surgery (Baseline) | | | Reaming ⌀ 13 mm | | 0.007217 | 0.00868 | 1.0000 |
| Before surgery (Baseline) | | | Reaming ⌀ 13.5 mm | | 0.006117 | 0.00868 | 1.0000 |
| Before surgery (Baseline) | | | Reaming ⌀ 14 mm | | 0.002283 | 0.00868 | 1.0000 |
| Before surgery (Baseline) | | | Reaming ⌀ 14.5 mm | | 0.009378 | 0.00913 | 0.9996 |
| Before surgery (Baseline) | | | Reaming ⌀ 15 mm | | 0.004418 | 0.00913 | 1.0000 |
| Before surgery (Baseline) | | | Reaming ⌀ 15.5 mm | | 0.01014 | 0.00913 | 0.9991 |
| Before surgery (Baseline) | | | Completion surgery +5 min | | 0.010417 | 0.00868 | 0.9979 |
| Opening femur | | | Harvesting bone marrow | | NA | NA | NA |
| Opening femur | | | Reaming ⌀ 10 mm | | 0.005628 | 0.00977 | 1.0000 |
| Opening femur | | | Reaming ⌀ 10.5 mm | | 0.000994 | 0.01248 | 1.0000 |
| Opening femur | | | Reaming ⌀ 11 mm | | 0.005653 | 0.00977 | 1.0000 |
| Opening femur | | | Reaming ⌀ 11.5 mm | | 0.005094 | 0.01248 | 1.0000 |
| Opening femur | | | Reaming ⌀ 12 mm | | 0.005928 | 0.00977 | 1.0000 |
| Opening femur | | | Reaming ⌀ 12.5 mm | | 0.002933 | 0.00868 | 1.0000 |
| Opening femur | | | Reaming ⌀ 13 mm | | 0.0018 | 0.00868 | 1.0000 |
| Opening femur | | | Reaming ⌀ 13.5 mm | | 0.0007 | 0.00868 | 1.0000 |
| Opening femur | | | Reaming ⌀ 14 mm | | -0.003133 | 0.00868 | 1.0000 |
| Opening femur | | | Reaming ⌀ 14.5 mm | | 0.003961 | 0.00913 | 1.0000 |
| Opening femur | | | Reaming ⌀ 15 mm | | -0.000999 | 0.00913 | 1.0000 |
| Opening femur | | | Reaming ⌀ 15.5 mm | | 0.004723 | 0.00913 | 1.0000 |
| Opening femur | | | Completion surgery +5 min | | 0.005 | 0.00868 | 1.0000 |
| Harvesting bone marrow | | | Reaming ⌀ 10 mm | | NA | NA | NA |
| Harvesting bone marrow | | | Reaming ⌀ 10.5 mm | | NA | NA | NA |
| Harvesting bone marrow | | | Reaming ⌀ 11 mm | | NA | NA | NA |
| Harvesting bone marrow | | | Reaming ⌀ 11.5 mm | | NA | NA | NA |
| Harvesting bone marrow | | | Reaming ⌀ 12 mm | | NA | NA | NA |
| Harvesting bone marrow | | | Reaming ⌀ 12.5 mm | | NA | NA | NA |
| Harvesting bone marrow | | | Reaming ⌀ 13 mm | | NA | NA | NA |
| Harvesting bone marrow | | | Reaming ⌀ 13.5 mm | | NA | NA | NA |
| Harvesting bone marrow | | | Reaming ⌀ 14 mm | | NA | NA | NA |
| Harvesting bone marrow | | | Reaming ⌀ 14.5 mm | | NA | NA | NA |
| Harvesting bone marrow | | | Reaming ⌀ 15 mm | | NA | NA | NA |
| Harvesting bone marrow | | | Reaming ⌀ 15.5 mm | | NA | NA | NA |
| Harvesting bone marrow | | | Completion surgery +5 min | | NA | NA | NA |
| Reaming ⌀ 10 mm | | | Reaming ⌀ 10.5 mm | | -0.004634 | 0.01345 | 1.0000 |
| Reaming ⌀ 10 mm | | | Reaming ⌀ 11 mm | | 0.000025 | 0.01063 | 1.0000 |
| Reaming ⌀ 10 mm | | | Reaming ⌀ 11.5 mm | | -0.000534 | 0.01345 | 1.0000 |
| Reaming ⌀ 10 mm | | | Reaming ⌀ 12 mm | | 0.0003 | 0.01063 | 1.0000 |
| Reaming ⌀ 10 mm | | | Reaming ⌀ 12.5 mm | | -0.002695 | 0.00977 | 1.0000 |
| Reaming ⌀ 10 mm | | | Reaming ⌀ 13 mm | | -0.003828 | 0.00977 | 1.0000 |
| Reaming ⌀ 10 mm | | | Reaming ⌀ 13.5 mm | | -0.004928 | 0.00977 | 1.0000 |
| Reaming ⌀ 10 mm | | | Reaming ⌀ 14 mm | | -0.008761 | 0.00977 | 0.9999 |
| Reaming ⌀ 10 mm | | | Reaming ⌀ 14.5 mm | | -0.001667 | 0.01019 | 1.0000 |
| Reaming ⌀ 10 mm | | | Reaming ⌀ 15 mm | | -0.006627 | 0.01012 | 1.0000 |
| Reaming ⌀ 10 mm | | | Reaming ⌀ 15.5 mm | | -0.000905 | 0.01019 | 1.0000 |
| Reaming ⌀ 10 mm | | | Completion surgery +5 min | | -0.000628 | 0.00977 | 1.0000 |
| Reaming ⌀ 10.5 mm | | | Reaming ⌀ 11 mm | | 0.004659 | 0.01345 | 1.0000 |
| Reaming ⌀ 10.5 mm | | | Reaming ⌀ 11.5 mm | | 0.0041 | 0.01503 | 1.0000 |
| Reaming ⌀ 10.5 mm | | | Reaming ⌀ 12 mm | | 0.004934 | 0.01345 | 1.0000 |
| Reaming ⌀ 10.5 mm | | | Reaming ⌀ 12.5 mm | | 0.001939 | 0.01248 | 1.0000 |
| Reaming ⌀ 10.5 mm | | | Reaming ⌀ 13 mm | | 0.000806 | 0.01248 | 1.0000 |
| Reaming ⌀ 10.5 mm | | | Reaming ⌀ 13.5 mm | | -0.000294 | 0.01248 | 1.0000 |
| Reaming ⌀ 10.5 mm | | | Reaming ⌀ 14 mm | | -0.004127 | 0.01248 | 1.0000 |
| Reaming ⌀ 10.5 mm | | | Reaming ⌀ 14.5 mm | | 0.002967 | 0.01275 | 1.0000 |
| Reaming ⌀ 10.5 mm | | | Reaming ⌀ 15 mm | | -0.001993 | 0.01288 | 1.0000 |
| Reaming ⌀ 10.5 mm | | | Reaming ⌀ 15.5 mm | | 0.003729 | 0.01275 | 1.0000 |
| Reaming ⌀ 10.5 mm | | | Completion surgery +5 min | | 0.004006 | 0.01248 | 1.0000 |
| Reaming ⌀ 11 mm | | | Reaming ⌀ 11.5 mm | | -0.000559 | 0.01345 | 1.0000 |
| Reaming ⌀ 11 mm | | | Reaming ⌀ 12 mm | | 0.000275 | 0.01063 | 1.0000 |
| Reaming ⌀ 11 mm | | | Reaming ⌀ 12.5 mm | | -0.00272 | 0.00977 | 1.0000 |
| Reaming ⌀ 11 mm | | | Reaming ⌀ 13 mm | | -0.003853 | 0.00977 | 1.0000 |
| Reaming ⌀ 11 mm | | | Reaming ⌀ 13.5 mm | | -0.004953 | 0.00977 | 1.0000 |
| Reaming ⌀ 11 mm | | | Reaming ⌀ 14 mm | | -0.008786 | 0.00977 | 0.9999 |
| Reaming ⌀ 11 mm | | | Reaming ⌀ 14.5 mm | | -0.001692 | 0.01019 | 1.0000 |
| Reaming ⌀ 11 mm | | | Reaming ⌀ 15 mm | | -0.006652 | 0.01012 | 1.0000 |
| Reaming ⌀ 11 mm | | | Reaming ⌀ 15.5 mm | | -0.00093 | 0.01019 | 1.0000 |
| Reaming ⌀ 11 mm | | | Completion surgery +5 min | | -0.000653 | 0.00977 | 1.0000 |
| Reaming ⌀ 11.5 mm | | | Reaming ⌀ 12 mm | | 0.000834 | 0.01345 | 1.0000 |
| Reaming ⌀ 11.5 mm | | | Reaming ⌀ 12.5 mm | | -0.002161 | 0.01248 | 1.0000 |
| Reaming ⌀ 11.5 mm | | | Reaming ⌀ 13 mm | | -0.003294 | 0.01248 | 1.0000 |
| Reaming ⌀ 11.5 mm | | | Reaming ⌀ 13.5 mm | | -0.004394 | 0.01248 | 1.0000 |
| Reaming ⌀ 11.5 mm | | | Reaming ⌀ 14 mm | | -0.008227 | 0.01248 | 1.0000 |
| Reaming ⌀ 11.5 mm | | | Reaming ⌀ 14.5 mm | | -0.001133 | 0.01275 | 1.0000 |
| Reaming ⌀ 11.5 mm | | | Reaming ⌀ 15 mm | | -0.006093 | 0.01288 | 1.0000 |
| Reaming ⌀ 11.5 mm | | | Reaming ⌀ 15.5 mm | | -0.000371 | 0.01275 | 1.0000 |
| Reaming ⌀ 11.5 mm | | | Completion surgery +5 min | | -0.000094 | 0.01248 | 1.0000 |
| Reaming ⌀ 12 mm | | | Reaming ⌀ 12.5 mm | | -0.002995 | 0.00977 | 1.0000 |
| Reaming ⌀ 12 mm | | | Reaming ⌀ 13 mm | | -0.004128 | 0.00977 | 1.0000 |
| Reaming ⌀ 12 mm | | | Reaming ⌀ 13.5 mm | | -0.005228 | 0.00977 | 1.0000 |
| Reaming ⌀ 12 mm | | | Reaming ⌀ 14 mm | | -0.009061 | 0.00977 | 0.9999 |
| Reaming ⌀ 12 mm | | | Reaming ⌀ 14.5 mm | | -0.001967 | 0.01019 | 1.0000 |
| Reaming ⌀ 12 mm | | | Reaming ⌀ 15 mm | | -0.006927 | 0.01012 | 1.0000 |
| Reaming ⌀ 12 mm | | | Reaming ⌀ 15.5 mm | | -0.001205 | 0.01019 | 1.0000 |
| Reaming ⌀ 12 mm | | | Completion surgery +5 min | | -0.000928 | 0.00977 | 1.0000 |
| Reaming ⌀ 12.5 mm | | | Reaming ⌀ 13 mm | | -0.001133 | 0.00868 | 1.0000 |
| Reaming ⌀ 12.5 mm | | | Reaming ⌀ 13.5 mm | | -0.002233 | 0.00868 | 1.0000 |
| Reaming ⌀ 12.5 mm | | | Reaming ⌀ 14 mm | | -0.006067 | 0.00868 | 1.0000 |
| Reaming ⌀ 12.5 mm | | | Reaming ⌀ 14.5 mm | | 0.001028 | 0.00913 | 1.0000 |
| Reaming ⌀ 12.5 mm | | | Reaming ⌀ 15 mm | | -0.003932 | 0.00913 | 1.0000 |
| Reaming ⌀ 12.5 mm | | | Reaming ⌀ 15.5 mm | | 0.00179 | 0.00913 | 1.0000 |
| Reaming ⌀ 12.5 mm | | | Completion surgery +5 min | | 0.002067 | 0.00868 | 1.0000 |
| Reaming ⌀ 13 mm | | | Reaming ⌀ 13.5 mm | | -0.0011 | 0.00868 | 1.0000 |
| Reaming ⌀ 13 mm | | | Reaming ⌀ 14 mm | | -0.004933 | 0.00868 | 1.0000 |
| Reaming ⌀ 13 mm | | | Reaming ⌀ 14.5 mm | | 0.002161 | 0.00913 | 1.0000 |
| Reaming ⌀ 13 mm | | | Reaming ⌀ 15 mm | | -0.002799 | 0.00913 | 1.0000 |
| Reaming ⌀ 13 mm | | | Reaming ⌀ 15.5 mm | | 0.002923 | 0.00913 | 1.0000 |
| Reaming ⌀ 13 mm | | | Completion surgery +5 min | | 0.0032 | 0.00868 | 1.0000 |
| Reaming ⌀ 13.5 mm | | | Reaming ⌀ 14 mm | | -0.003833 | 0.00868 | 1.0000 |
| Reaming ⌀ 13.5 mm | | | Reaming ⌀ 14.5 mm | | 0.003261 | 0.00913 | 1.0000 |
| Reaming ⌀ 13.5 mm | | | Reaming ⌀ 15 mm | | -0.001699 | 0.00913 | 1.0000 |
| Reaming ⌀ 13.5 mm | | | Reaming ⌀ 15.5 mm | | 0.004023 | 0.00913 | 1.0000 |
| Reaming ⌀ 13.5 mm | | | Completion surgery +5 min | | 0.0043 | 0.00868 | 1.0000 |
| Reaming ⌀ 14 mm | | | Reaming ⌀ 14.5 mm | | 0.007095 | 0.00913 | 1.0000 |
| Reaming ⌀ 14 mm | | | Reaming ⌀ 15 mm | | 0.002134 | 0.00913 | 1.0000 |
| Reaming ⌀ 14 mm | | | Reaming ⌀ 15.5 mm | | 0.007856 | 0.00913 | 1.0000 |
| Reaming ⌀ 14 mm | | | Completion surgery +5 min | | 0.008133 | 0.00868 | 0.9999 |
| Reaming ⌀ 14.5 mm | | | Reaming ⌀ 15 mm | | -0.00496 | 0.00957 | 1.0000 |
| Reaming ⌀ 14.5 mm | | | Reaming ⌀ 15.5 mm | | 0.000762 | 0.00956 | 1.0000 |
| Reaming ⌀ 14.5 mm | | | Completion surgery +5 min | | 0.001039 | 0.00913 | 1.0000 |
| Reaming ⌀ 15 mm | | | Reaming ⌀ 15.5 mm | | 0.005722 | 0.00957 | 1.0000 |
| Reaming ⌀ 15 mm | | | Completion surgery +5 min | | 0.005999 | 0.00913 | 1.0000 |
| Reaming ⌀ 15.5 mm | | | Completion surgery +5 min | | 0.000277 | 0.00913 | 1.0000 |
| **A+R+A group** | | | | | | | |
| **Time points** | | | | | **Estimated mean difference** | **Standard error** | **P - value** |
| Before surgery (Baseline) | | | Opening femur | | 0.01006 | 0.00951 | 0.9995 |
| Before surgery (Baseline) | | | Harvesting bone marrow | | 0.005173 | 0.00913 | 1.0000 |
| Before surgery (Baseline) | | | Reaming ⌀ 10 mm | | 0.015738 | 0.01012 | 0.9725 |
| Before surgery (Baseline) | | | Reaming ⌀ 10.5 mm | | -0.003759 | 0.0129 | 1.0000 |
| Before surgery (Baseline) | | | Reaming ⌀ 11 mm | | 0.016538 | 0.01012 | 0.9580 |
| Before surgery (Baseline) | | | Reaming ⌀ 11.5 mm | | 0.016247 | 0.01681 | 0.9998 |
| Before surgery (Baseline) | | | Reaming ⌀ 12 mm | | 0.011788 | 0.01012 | 0.9985 |
| Before surgery (Baseline) | | | Reaming ⌀ 12.5 mm | | 0.01902 | 0.00951 | 0.8190 |
| Before surgery (Baseline) | | | Reaming ⌀ 13 mm | | 0.019955 | 0.00958 | 0.7709 |
| Before surgery (Baseline) | | | Reaming ⌀ 13.5 mm | | 0.00358 | 0.00951 | 1.0000 |
| Before surgery (Baseline) | | | Reaming ⌀ 14 mm | | 0.015435 | 0.00958 | 0.9627 |
| Before surgery (Baseline) | | | Reaming ⌀ 14.5 mm | | 0.017356 | 0.00913 | 0.8698 |
| Before surgery (Baseline) | | | Reaming ⌀ 15 mm | | 0.01876 | 0.01012 | 0.8900 |
| Before surgery (Baseline) | | | Reaming ⌀ 15.5 mm | | 0.021036 | 0.01106 | 0.8692 |
| Before surgery (Baseline) | | | Completion surgery +5 min | | 0.014789 | 0.00913 | 0.9612 |
| Opening femur | | | Harvesting bone marrow | | -0.004887 | 0.00913 | 1.0000 |
| Opening femur | | | Reaming ⌀ 10 mm | | 0.005678 | 0.01012 | 1.0000 |
| Opening femur | | | Reaming ⌀ 10.5 mm | | -0.013819 | 0.0129 | 0.9994 |
| Opening femur | | | Reaming ⌀ 11 mm | | 0.006478 | 0.01012 | 1.0000 |
| Opening femur | | | Reaming ⌀ 11.5 mm | | 0.006187 | 0.01681 | 1.0000 |
| Opening femur | | | Reaming ⌀ 12 mm | | 0.001728 | 0.01012 | 1.0000 |
| Opening femur | | | Reaming ⌀ 12.5 mm | | 0.00896 | 0.00951 | 0.9999 |
| Opening femur | | | Reaming ⌀ 13 mm | | 0.009895 | 0.00958 | 0.9996 |
| Opening femur | | | Reaming ⌀ 13.5 mm | | -0.00648 | 0.00951 | 1.0000 |
| Opening femur | | | Reaming ⌀ 14 mm | | 0.005375 | 0.00958 | 1.0000 |
| Opening femur | | | Reaming ⌀ 14.5 mm | | 0.007296 | 0.00913 | 1.0000 |
| Opening femur | | | Reaming ⌀ 15 mm | | 0.0087 | 0.01012 | 1.0000 |
| Opening femur | | | Reaming ⌀ 15.5 mm | | 0.010976 | 0.01106 | 0.9998 |
| Opening femur | | | Completion surgery +5 min | | 0.004729 | 0.00913 | 1.0000 |
| Harvesting bone marrow | | | Reaming ⌀ 10 mm | | 0.010566 | 0.00977 | 0.9994 |
| Harvesting bone marrow | | | Reaming ⌀ 10.5 mm | | -0.008932 | 0.01249 | 1.0000 |
| Harvesting bone marrow | | | Reaming ⌀ 11 mm | | 0.011366 | 0.00977 | 0.9985 |
| Harvesting bone marrow | | | Reaming ⌀ 11.5 mm | | 0.011074 | 0.0166 | 1.0000 |
| Harvesting bone marrow | | | Reaming ⌀ 12 mm | | 0.006616 | 0.00977 | 1.0000 |
| Harvesting bone marrow | | | Reaming ⌀ 12.5 mm | | 0.013847 | 0.00913 | 0.9781 |
| Harvesting bone marrow | | | Reaming ⌀ 13 mm | | 0.014782 | 0.00913 | 0.9611 |
| Harvesting bone marrow | | | Reaming ⌀ 13.5 mm | | -0.001593 | 0.00913 | 1.0000 |
| Harvesting bone marrow | | | Reaming ⌀ 14 mm | | 0.010262 | 0.00913 | 0.9990 |
| Harvesting bone marrow | | | Reaming ⌀ 14.5 mm | | 0.012183 | 0.00868 | 0.9894 |
| Harvesting bone marrow | | | Reaming ⌀ 15 mm | | 0.013587 | 0.00977 | 0.9903 |
| Harvesting bone marrow | | | Reaming ⌀ 15.5 mm | | 0.015863 | 0.01074 | 0.9828 |
| Harvesting bone marrow | | | Completion surgery +5 min | | 0.009617 | 0.00868 | 0.9991 |
| Reaming ⌀ 10 mm | | | Reaming ⌀ 10.5 mm | | -0.019497 | 0.01347 | 0.9859 |
| Reaming ⌀ 10 mm | | | Reaming ⌀ 11 mm | | 0.0008 | 0.01063 | 1.0000 |
| Reaming ⌀ 10 mm | | | Reaming ⌀ 11.5 mm | | 0.000508 | 0.01733 | 1.0000 |
| Reaming ⌀ 10 mm | | | Reaming ⌀ 12 mm | | -0.00395 | 0.01063 | 1.0000 |
| Reaming ⌀ 10 mm | | | Reaming ⌀ 12.5 mm | | 0.003282 | 0.01012 | 1.0000 |
| Reaming ⌀ 10 mm | | | Reaming ⌀ 13 mm | | 0.004217 | 0.0102 | 1.0000 |
| Reaming ⌀ 10 mm | | | Reaming ⌀ 13.5 mm | | -0.012158 | 0.01012 | 0.9979 |
| Reaming ⌀ 10 mm | | | Reaming ⌀ 14 mm | | -0.000303 | 0.0102 | 1.0000 |
| Reaming ⌀ 10 mm | | | Reaming ⌀ 14.5 mm | | 0.001618 | 0.00977 | 1.0000 |
| Reaming ⌀ 10 mm | | | Reaming ⌀ 15 mm | | 0.003022 | 0.01072 | 1.0000 |
| Reaming ⌀ 10 mm | | | Reaming ⌀ 15.5 mm | | 0.005298 | 0.01164 | 1.0000 |
| Reaming ⌀ 10 mm | | | Completion surgery +5 min | | -0.000949 | 0.00977 | 1.0000 |
| Reaming ⌀ 10.5 mm | | | Reaming ⌀ 11 mm | | 0.020297 | 0.01347 | 0.9795 |
| Reaming ⌀ 10.5 mm | | | Reaming ⌀ 11.5 mm | | 0.020006 | 0.01862 | 0.9994 |
| Reaming ⌀ 10.5 mm | | | Reaming ⌀ 12 mm | | 0.015547 | 0.01347 | 0.9987 |
| Reaming ⌀ 10.5 mm | | | Reaming ⌀ 12.5 mm | | 0.022779 | 0.0129 | 0.9230 |
| Reaming ⌀ 10.5 mm | | | Reaming ⌀ 13 mm | | 0.023714 | 0.01276 | 0.8882 |
| Reaming ⌀ 10.5 mm | | | Reaming ⌀ 13.5 mm | | 0.007339 | 0.0129 | 1.0000 |
| Reaming ⌀ 10.5 mm | | | Reaming ⌀ 14 mm | | 0.019194 | 0.01276 | 0.9797 |
| Reaming ⌀ 10.5 mm | | | Reaming ⌀ 14.5 mm | | 0.021115 | 0.01249 | 0.9448 |
| Reaming ⌀ 10.5 mm | | | Reaming ⌀ 15 mm | | 0.022519 | 0.01333 | 0.9451 |
| Reaming ⌀ 10.5 mm | | | Reaming ⌀ 15.5 mm | | 0.024795 | 0.01401 | 0.9216 |
| Reaming ⌀ 10.5 mm | | | Completion surgery +5 min | | 0.018548 | 0.01249 | 0.9820 |
| Reaming ⌀ 11 mm | | | Reaming ⌀ 11.5 mm | | -0.000292 | 0.01733 | 1.0000 |
| Reaming ⌀ 11 mm | | | Reaming ⌀ 12 mm | | -0.00475 | 0.01063 | 1.0000 |
| Reaming ⌀ 11 mm | | | Reaming ⌀ 12.5 mm | | 0.002482 | 0.01012 | 1.0000 |
| Reaming ⌀ 11 mm | | | Reaming ⌀ 13 mm | | 0.003417 | 0.0102 | 1.0000 |
| Reaming ⌀ 11 mm | | | Reaming ⌀ 13.5 mm | | -0.012958 | 0.01012 | 0.9958 |
| Reaming ⌀ 11 mm | | | Reaming ⌀ 14 mm | | -0.001103 | 0.0102 | 1.0000 |
| Reaming ⌀ 11 mm | | | Reaming ⌀ 14.5 mm | | 0.000818 | 0.00977 | 1.0000 |
| Reaming ⌀ 11 mm | | | Reaming ⌀ 15 mm | | 0.002222 | 0.01072 | 1.0000 |
| Reaming ⌀ 11 mm | | | Reaming ⌀ 15.5 mm | | 0.004498 | 0.01164 | 1.0000 |
| Reaming ⌀ 11 mm | | | Completion surgery +5 min | | -0.001749 | 0.00977 | 1.0000 |
| Reaming ⌀ 11.5 mm | | | Reaming ⌀ 12 mm | | -0.004458 | 0.01733 | 1.0000 |
| Reaming ⌀ 11.5 mm | | | Reaming ⌀ 12.5 mm | | 0.002773 | 0.01681 | 1.0000 |
| Reaming ⌀ 11.5 mm | | | Reaming ⌀ 13 mm | | 0.003708 | 0.0168 | 1.0000 |
| Reaming ⌀ 11.5 mm | | | Reaming ⌀ 13.5 mm | | -0.012667 | 0.01681 | 1.0000 |
| Reaming ⌀ 11.5 mm | | | Reaming ⌀ 14 mm | | -0.000812 | 0.0168 | 1.0000 |
| Reaming ⌀ 11.5 mm | | | Reaming ⌀ 14.5 mm | | 0.001109 | 0.0166 | 1.0000 |
| Reaming ⌀ 11.5 mm | | | Reaming ⌀ 15 mm | | 0.002513 | 0.01711 | 1.0000 |
| Reaming ⌀ 11.5 mm | | | Reaming ⌀ 15.5 mm | | 0.004789 | 0.01762 | 1.0000 |
| Reaming ⌀ 11.5 mm | | | Completion surgery +5 min | | -0.001457 | 0.0166 | 1.0000 |
| Reaming ⌀ 12 mm | | | Reaming ⌀ 12.5 mm | | 0.007232 | 0.01012 | 1.0000 |
| Reaming ⌀ 12 mm | | | Reaming ⌀ 13 mm | | 0.008167 | 0.0102 | 1.0000 |
| Reaming ⌀ 12 mm | | | Reaming ⌀ 13.5 mm | | -0.008208 | 0.01012 | 1.0000 |
| Reaming ⌀ 12 mm | | | Reaming ⌀ 14 mm | | 0.003647 | 0.0102 | 1.0000 |
| Reaming ⌀ 12 mm | | | Reaming ⌀ 14.5 mm | | 0.005568 | 0.00977 | 1.0000 |
| Reaming ⌀ 12 mm | | | Reaming ⌀ 15 mm | | 0.006972 | 0.01072 | 1.0000 |
| Reaming ⌀ 12 mm | | | Reaming ⌀ 15.5 mm | | 0.009248 | 0.01164 | 1.0000 |
| Reaming ⌀ 12 mm | | | Completion surgery +5 min | | 0.003001 | 0.00977 | 1.0000 |
| Reaming ⌀ 12.5 mm | | | Reaming ⌀ 13 mm | | 0.000935 | 0.00958 | 1.0000 |
| Reaming ⌀ 12.5 mm | | | Reaming ⌀ 13.5 mm | | -0.01544 | 0.00951 | 0.9601 |
| Reaming ⌀ 12.5 mm | | | Reaming ⌀ 14 mm | | -0.003585 | 0.00958 | 1.0000 |
| Reaming ⌀ 12.5 mm | | | Reaming ⌀ 14.5 mm | | -0.001664 | 0.00913 | 1.0000 |
| Reaming ⌀ 12.5 mm | | | Reaming ⌀ 15 mm | | -0.00026 | 0.01012 | 1.0000 |
| Reaming ⌀ 12.5 mm | | | Reaming ⌀ 15.5 mm | | 0.002016 | 0.01106 | 1.0000 |
| Reaming ⌀ 12.5 mm | | | Completion surgery +5 min | | -0.004231 | 0.00913 | 1.0000 |
| Reaming ⌀ 13 mm | | | Reaming ⌀ 13.5 mm | | -0.016375 | 0.00958 | 0.9396 |
| Reaming ⌀ 13 mm | | | Reaming ⌀ 14 mm | | -0.00452 | 0.00951 | 1.0000 |
| Reaming ⌀ 13 mm | | | Reaming ⌀ 14.5 mm | | -0.002599 | 0.00913 | 1.0000 |
| Reaming ⌀ 13 mm | | | Reaming ⌀ 15 mm | | -0.001195 | 0.01013 | 1.0000 |
| Reaming ⌀ 13 mm | | | Reaming ⌀ 15.5 mm | | 0.001081 | 0.01107 | 1.0000 |
| Reaming ⌀ 13 mm | | | Completion surgery +5 min | | -0.005166 | 0.00913 | 1.0000 |
| Reaming ⌀ 13.5 mm | | | Reaming ⌀ 14 mm | | 0.011855 | 0.00958 | 0.9971 |
| Reaming ⌀ 13.5 mm | | | Reaming ⌀ 14.5 mm | | 0.013776 | 0.00913 | 0.9792 |
| Reaming ⌀ 13.5 mm | | | Reaming ⌀ 15 mm | | 0.01518 | 0.01012 | 0.9801 |
| Reaming ⌀ 13.5 mm | | | Reaming ⌀ 15.5 mm | | 0.017456 | 0.01106 | 0.9688 |
| Reaming ⌀ 13.5 mm | | | Completion surgery +5 min | | 0.011209 | 0.00913 | 0.9973 |
| Reaming ⌀ 14 mm | | | Reaming ⌀ 14.5 mm | | 0.001921 | 0.00913 | 1.0000 |
| Reaming ⌀ 14 mm | | | Reaming ⌀ 15 mm | | 0.003325 | 0.01013 | 1.0000 |
| Reaming ⌀ 14 mm | | | Reaming ⌀ 15.5 mm | | 0.005601 | 0.01107 | 1.0000 |
| Reaming ⌀ 14 mm | | | Completion surgery +5 min | | -0.000646 | 0.00913 | 1.0000 |
| Reaming ⌀ 14.5 mm | | | Reaming ⌀ 15 mm | | 0.001404 | 0.00977 | 1.0000 |
| Reaming ⌀ 14.5 mm | | | Reaming ⌀ 15.5 mm | | 0.00368 | 0.01074 | 1.0000 |
| Reaming ⌀ 14.5 mm | | | Completion surgery +5 min | | -0.002567 | 0.00868 | 1.0000 |
| Reaming ⌀ 15 mm | | | Reaming ⌀ 15.5 mm | | 0.002276 | 0.01153 | 1.0000 |
| Reaming ⌀ 15 mm | | | Completion surgery +5 min | | -0.003971 | 0.00977 | 1.0000 |
| Reaming ⌀ 15.5 mm | | | Completion surgery +5 min | | -0.006247 | 0.01074 | 1.0000 |

## Suppl. Table 2. Average Gurd rating compared between experimental groups per time point and within group over different time points.

|  | **RIA 2 group** | | | **A+R+A group** | | **Comparison experimental (RIA 2/A+R+A) groups** | |
| --- | --- | --- | --- | --- | --- | --- | --- |
| **Time point** | **Estimated mean (standard error)** | | **95% confidence interval (lower / upper)** | **Estimated mean (standard error)** | **95% confidence interval (lower / upper)** | **Estimated mean difference (standard error** | **P - value** |
| Before surgery (Baseline) | 0.583 (0.210) | | (0.1673 / 0.999) | 0.800 (0.230) | (0.3435 / 1.256) | -0.2167 (0.312) | 0.4884 |
| Opening femur | 0.833 (0.210) | | (0.4173 / 1.249) | 0.700 (0.230) | (0.2435 / 1.156) | 0.1333 (0.312) | 0.6697 |
| Harvesting bone marrow | NA | | NA | 0.833 (0.210) | (0.4173 / 1.249) | NA | NA |
| Reaming ⌀ 10 mm | 0.500 (0.259) | | (-0.0122 / 1.012) | 0.875 (0.258) | (0.3631 / 1.387) | -0.3750 (0.365) | 0.3071 |
| Reaming ⌀ 10.5 mm | 0.500 (0.367) | | (-0.2281 / 1.228) | 0.750 (0.367) | (0.0228 / 1.477) | -0.2500 (0.519) | 0.6312 |
| Reaming ⌀ 11 mm | 0.625 (0.259) | | (0.1128 / 1.137) | 1.000 (0.258) | (0.4881 / 1.512) | -0.3750 (0.365) | 0.3071 |
| Reaming ⌀ 11.5 mm | 0.750 (0.367) | | (0.0219 / 1.478) | 0.500 (0.522) | (-0.5347 / 1.535) | 0.2500 (0.639) | 0.6962 |
| Reaming ⌀ 12 mm | 0.375 (0.259) | | (-0.1372 / 0.887) | 1.000 (0.258) | (0.4881 / 1.512) | -0.6250 (0.365) | 0.0900 |
| Reaming ⌀ 12.5 mm | 0.583 (0.210) | | (0.1673 / 0.999) | 0.500 (0.230) | (0.0435 / 0.956) | 0.0833 (0.312) | 0.7897 |
| Reaming ⌀ 13 mm | 0.583 (0.210) | | (0.1673 / 0.999) | 0.500 (0.231) | (0.0430 / 0.957) | 0.0833 (0.312) | 0.7898 |
| Reaming ⌀ 13.5 mm | 0.667 (0.210) | | (0.2506 / 1.083) | 0.700 (0.230) | (0.2435 / 1.156) | -0.0333 (0.312) | 0.9150 |
| Reaming ⌀ 14 mm | 0.667 (0.210) | | (0.2506 / 1.083) | 0.900 (0.231) | (0.4430 / 1.357) | -0.2333 (0.312) | 0.4560 |
| Reaming ⌀ 14.5 mm | 0.600 (0.231) | | (0.1428 / 1.057) | 0.750 (0.210) | (0.3340 / 1.166) | -0.1500 (0.312) | 0.6316 |
| Reaming ⌀ 15 mm | 0.800 (0.231) | | (0.3430 / 1.257) | 0.500 (0.258) | (-0.0120 / 1.012) | 0.3000 (0.346) | 0.3883 |
| Reaming ⌀ 15.5 mm | 0.800 (0.231) | | (0.3428 / 1.257) | 0.500 (0.300) | (-0.0936 / 1.094) | 0.3000 (0.378) | 0.4293 |
| Completion surgery +5 min | 0.583 (0.210) | | (0.1673 / 0.999) | 0.750 (0.210) | (0.3340 / 1.166) | -0.1667 (0.297) | 0.5757 |
| **RIA 2 group** | | | | | | | |
| **Time points** | | | | | **Estimated mean difference** | **Standard error** | **P - value** |
| Before surgery (Baseline) | | Opening femur | | | -0.2500 | 0.297 | 1.0000 |
| Before surgery (Baseline) | | Harvesting bone marrow | | | NA | NA | NA |
| Before surgery (Baseline) | | Reaming ⌀ 10 mm | | | 0.0833 | 0.333 | 1.0000 |
| Before surgery (Baseline) | | Reaming ⌀ 10.5 mm | | | 0.0833 | 0.423 | 1.0000 |
| Before surgery (Baseline) | | Reaming ⌀ 11 mm | | | -0.0417 | 0.333 | 1.0000 |
| Before surgery (Baseline) | | Reaming ⌀ 11.5 mm | | | -0.1667 | 0.423 | 1.0000 |
| Before surgery (Baseline) | | Reaming ⌀ 12 mm | | | 0.2083 | 0.333 | 1.0000 |
| Before surgery (Baseline) | | Reaming ⌀ 12.5 mm | | | 0.0000 | 0.297 | 1.0000 |
| Before surgery (Baseline) | | Reaming ⌀ 13 mm | | | 0.0000 | 0.297 | 1.0000 |
| Before surgery (Baseline) | | Reaming ⌀ 13.5 mm | | | -0.0833 | 0.297 | 1.0000 |
| Before surgery (Baseline) | | Reaming ⌀ 14 mm | | | -0.0833 | 0.297 | 1.0000 |
| Before surgery (Baseline) | | Reaming ⌀ 14.5 mm | | | -0.0167 | 0.312 | 1.0000 |
| Before surgery (Baseline) | | Reaming ⌀ 15 mm | | | -0.2167 | 0.312 | 1.0000 |
| Before surgery (Baseline) | | Reaming ⌀ 15.5 mm | | | -0.2167 | 0.312 | 1.0000 |
| Before surgery (Baseline) | | Completion surgery +5 min | | | 0.0000 | 0.297 | 1.0000 |
| Opening femur | | Harvesting bone marrow | | | NA | NA | NA |
| Opening femur | | Reaming ⌀ 10 mm | | | 0.3333 | 0.333 | 0.9997 |
| Opening femur | | Reaming ⌀ 10.5 mm | | | 0.3333 | 0.423 | 1.0000 |
| Opening femur | | Reaming ⌀ 11 mm | | | 0.2083 | 0.333 | 1.0000 |
| Opening femur | | Reaming ⌀ 11.5 mm | | | 0.0833 | 0.423 | 1.0000 |
| Opening femur | | Reaming ⌀ 12 mm | | | 0.4583 | 0.333 | 0.9913 |
| Opening femur | | Reaming ⌀ 12.5 mm | | | 0.2500 | 0.297 | 1.0000 |
| Opening femur | | Reaming ⌀ 13 mm | | | 0.2500 | 0.297 | 1.0000 |
| Opening femur | | Reaming ⌀ 13.5 mm | | | 0.1667 | 0.297 | 1.0000 |
| Opening femur | | Reaming ⌀ 14 mm | | | 0.1667 | 0.297 | 1.0000 |
| Opening femur | | Reaming ⌀ 14.5 mm | | | 0.2333 | 0.312 | 1.0000 |
| Opening femur | | Reaming ⌀ 15 mm | | | 0.0333 | 0.312 | 1.0000 |
| Opening femur | | Reaming ⌀ 15.5 mm | | | 0.0333 | 0.312 | 1.0000 |
| Opening femur | | Completion surgery +5 min | | | 0.2500 | 0.297 | 1.0000 |
| Harvesting bone marrow | | Reaming ⌀ 10 mm | | | NA | NA | NA |
| Harvesting bone marrow | | Reaming ⌀ 10.5 mm | | | NA | NA | NA |
| Harvesting bone marrow | | Reaming ⌀ 11 mm | | | NA | NA | NA |
| Harvesting bone marrow | | Reaming ⌀ 11.5 mm | | | NA | NA | NA |
| Harvesting bone marrow | | Reaming ⌀ 12 mm | | | NA | NA | NA |
| Harvesting bone marrow | | Reaming ⌀ 12.5 mm | | | NA | NA | NA |
| Harvesting bone marrow | | Reaming ⌀ 13 mm | | | NA | NA | NA |
| Harvesting bone marrow | | Reaming ⌀ 13.5 mm | | | NA | NA | NA |
| Harvesting bone marrow | | Reaming ⌀ 14 mm | | | NA | NA | NA |
| Harvesting bone marrow | | Reaming ⌀ 14.5 mm | | | NA | NA | NA |
| Harvesting bone marrow | | Reaming ⌀ 15 mm | | | NA | NA | NA |
| Harvesting bone marrow | | Reaming ⌀ 15.5 mm | | | NA | NA | NA |
| Harvesting bone marrow | | Completion surgery +5 min | | | NA | NA | NA |
| Reaming ⌀ 10 mm | | Reaming ⌀ 10.5 mm | | | 0.0000 | 0.452 | 1.0000 |
| Reaming ⌀ 10 mm | | Reaming ⌀ 11 mm | | | -0.1250 | 0.364 | 1.0000 |
| Reaming ⌀ 10 mm | | Reaming ⌀ 11.5 mm | | | -0.2500 | 0.452 | 1.0000 |
| Reaming ⌀ 10 mm | | Reaming ⌀ 12 mm | | | 0.1250 | 0.364 | 1.0000 |
| Reaming ⌀ 10 mm | | Reaming ⌀ 12.5 mm | | | -0.0833 | 0.333 | 1.0000 |
| Reaming ⌀ 10 mm | | Reaming ⌀ 13 mm | | | -0.0833 | 0.333 | 1.0000 |
| Reaming ⌀ 10 mm | | Reaming ⌀ 13.5 mm | | | -0.1667 | 0.333 | 1.0000 |
| Reaming ⌀ 10 mm | | Reaming ⌀ 14 mm | | | -0.1667 | 0.333 | 1.0000 |
| Reaming ⌀ 10 mm | | Reaming ⌀ 14.5 mm | | | -0.1000 | 0.347 | 1.0000 |
| Reaming ⌀ 10 mm | | Reaming ⌀ 15 mm | | | -0.3000 | 0.346 | 1.0000 |
| Reaming ⌀ 10 mm | | Reaming ⌀ 15.5 mm | | | -0.3000 | 0.347 | 1.0000 |
| Reaming ⌀ 10 mm | | Completion surgery +5 min | | | -0.0833 | 0.333 | 1.0000 |
| Reaming ⌀ 10.5 mm | | Reaming ⌀ 11 mm | | | -0.1250 | 0.452 | 1.0000 |
| Reaming ⌀ 10.5 mm | | Reaming ⌀ 11.5 mm | | | -0.2500 | 0.514 | 1.0000 |
| Reaming ⌀ 10.5 mm | | Reaming ⌀ 12 mm | | | 0.1250 | 0.452 | 1.0000 |
| Reaming ⌀ 10.5 mm | | Reaming ⌀ 12.5 mm | | | -0.0833 | 0.423 | 1.0000 |
| Reaming ⌀ 10.5 mm | | Reaming ⌀ 13 mm | | | -0.0833 | 0.423 | 1.0000 |
| Reaming ⌀ 10.5 mm | | Reaming ⌀ 13.5 mm | | | -0.1667 | 0.423 | 1.0000 |
| Reaming ⌀ 10.5 mm | | Reaming ⌀ 14 mm | | | -0.1667 | 0.423 | 1.0000 |
| Reaming ⌀ 10.5 mm | | Reaming ⌀ 14.5 mm | | | -0.1000 | 0.433 | 1.0000 |
| Reaming ⌀ 10.5 mm | | Reaming ⌀ 15 mm | | | -0.3000 | 0.435 | 1.0000 |
| Reaming ⌀ 10.5 mm | | Reaming ⌀ 15.5 mm | | | -0.3000 | 0.433 | 1.0000 |
| Reaming ⌀ 10.5 mm | | Completion surgery +5 min | | | -0.0833 | 0.423 | 1.0000 |
| Reaming ⌀ 11 mm | | Reaming ⌀ 11.5 mm | | | -0.1250 | 0.452 | 1.0000 |
| Reaming ⌀ 11 mm | | Reaming ⌀ 12 mm | | | 0.2500 | 0.364 | 1.0000 |
| Reaming ⌀ 11 mm | | Reaming ⌀ 12.5 mm | | | 0.0417 | 0.333 | 1.0000 |
| Reaming ⌀ 11 mm | | Reaming ⌀ 13 mm | | | 0.0417 | 0.333 | 1.0000 |
| Reaming ⌀ 11 mm | | Reaming ⌀ 13.5 mm | | | -0.0417 | 0.333 | 1.0000 |
| Reaming ⌀ 11 mm | | Reaming ⌀ 14 mm | | | -0.0417 | 0.333 | 1.0000 |
| Reaming ⌀ 11 mm | | Reaming ⌀ 14.5 mm | | | 0.0250 | 0.347 | 1.0000 |
| Reaming ⌀ 11 mm | | Reaming ⌀ 15 mm | | | -0.1750 | 0.346 | 1.0000 |
| Reaming ⌀ 11 mm | | Reaming ⌀ 15.5 mm | | | -0.1750 | 0.347 | 1.0000 |
| Reaming ⌀ 11 mm | | Completion surgery +5 min | | | 0.0417 | 0.333 | 1.0000 |
| Reaming ⌀ 11.5 mm | | Reaming ⌀ 12 mm | | | 0.3750 | 0.452 | 1.0000 |
| Reaming ⌀ 11.5 mm | | Reaming ⌀ 12.5 mm | | | 0.1667 | 0.423 | 1.0000 |
| Reaming ⌀ 11.5 mm | | Reaming ⌀ 13 mm | | | 0.1667 | 0.423 | 1.0000 |
| Reaming ⌀ 11.5 mm | | Reaming ⌀ 13.5 mm | | | 0.0833 | 0.423 | 1.0000 |
| Reaming ⌀ 11.5 mm | | Reaming ⌀ 14 mm | | | 0.0833 | 0.423 | 1.0000 |
| Reaming ⌀ 11.5 mm | | Reaming ⌀ 14.5 mm | | | 0.1500 | 0.433 | 1.0000 |
| Reaming ⌀ 11.5 mm | | Reaming ⌀ 15 mm | | | -0.0500 | 0.435 | 1.0000 |
| Reaming ⌀ 11.5 mm | | Reaming ⌀ 15.5 mm | | | -0.0500 | 0.433 | 1.0000 |
| Reaming ⌀ 11.5 mm | | Completion surgery +5 min | | | 0.1667 | 0.423 | 1.0000 |
| Reaming ⌀ 12 mm | | Reaming ⌀ 12.5 mm | | | -0.2083 | 0.333 | 1.0000 |
| Reaming ⌀ 12 mm | | Reaming ⌀ 13 mm | | | -0.2083 | 0.333 | 1.0000 |
| Reaming ⌀ 12 mm | | Reaming ⌀ 13.5 mm | | | -0.2917 | 0.333 | 1.0000 |
| Reaming ⌀ 12 mm | | Reaming ⌀ 14 mm | | | -0.2917 | 0.333 | 1.0000 |
| Reaming ⌀ 12 mm | | Reaming ⌀ 14.5 mm | | | -0.2250 | 0.347 | 1.0000 |
| Reaming ⌀ 12 mm | | Reaming ⌀ 15 mm | | | -0.4250 | 0.346 | 0.9973 |
| Reaming ⌀ 12 mm | | Reaming ⌀ 15.5 mm | | | -0.4250 | 0.347 | 0.9974 |
| Reaming ⌀ 12 mm | | Completion surgery +5 min | | | -0.2083 | 0.333 | 1.0000 |
| Reaming ⌀ 12.5 mm | | Reaming ⌀ 13 mm | | | 0.0000 | 0.297 | 1.0000 |
| Reaming ⌀ 12.5 mm | | Reaming ⌀ 13.5 mm | | | -0.0833 | 0.297 | 1.0000 |
| Reaming ⌀ 12.5 mm | | Reaming ⌀ 14 mm | | | -0.0833 | 0.297 | 1.0000 |
| Reaming ⌀ 12.5 mm | | Reaming ⌀ 14.5 mm | | | -0.0167 | 0.312 | 1.0000 |
| Reaming ⌀ 12.5 mm | | Reaming ⌀ 15 mm | | | -0.2167 | 0.312 | 1.0000 |
| Reaming ⌀ 12.5 mm | | Reaming ⌀ 15.5 mm | | | -0.2167 | 0.312 | 1.0000 |
| Reaming ⌀ 12.5 mm | | Completion surgery +5 min | | | 0.0000 | 0.297 | 1.0000 |
| Reaming ⌀ 13 mm | | Reaming ⌀ 13.5 mm | | | -0.0833 | 0.297 | 1.0000 |
| Reaming ⌀ 13 mm | | Reaming ⌀ 14 mm | | | -0.0833 | 0.297 | 1.0000 |
| Reaming ⌀ 13 mm | | Reaming ⌀ 14.5 mm | | | -0.0167 | 0.312 | 1.0000 |
| Reaming ⌀ 13 mm | | Reaming ⌀ 15 mm | | | -0.2167 | 0.312 | 1.0000 |
| Reaming ⌀ 13 mm | | Reaming ⌀ 15.5 mm | | | -0.2167 | 0.312 | 1.0000 |
| Reaming ⌀ 13 mm | | Completion surgery +5 min | | | 0.0000 | 0.297 | 1.0000 |
| Reaming ⌀ 13.5 mm | | Reaming ⌀ 14 mm | | | 0.0000 | 0.297 | 1.0000 |
| Reaming ⌀ 13.5 mm | | Reaming ⌀ 14.5 mm | | | 0.0667 | 0.312 | 1.0000 |
| Reaming ⌀ 13.5 mm | | Reaming ⌀ 15 mm | | | -0.1333 | 0.312 | 1.0000 |
| Reaming ⌀ 13.5 mm | | Reaming ⌀ 15.5 mm | | | -0.1333 | 0.312 | 1.0000 |
| Reaming ⌀ 13.5 mm | | Completion surgery +5 min | | | 0.0833 | 0.297 | 1.0000 |
| Reaming ⌀ 14 mm | | Reaming ⌀ 14.5 mm | | | 0.0667 | 0.312 | 1.0000 |
| Reaming ⌀ 14 mm | | Reaming ⌀ 15 mm | | | -0.1333 | 0.312 | 1.0000 |
| Reaming ⌀ 14 mm | | Reaming ⌀ 15.5 mm | | | -0.1333 | 0.312 | 1.0000 |
| Reaming ⌀ 14 mm | | Completion surgery +5 min | | | 0.0833 | 0.297 | 1.0000 |
| Reaming ⌀ 14.5 mm | | Reaming ⌀ 15 mm | | | -0.2000 | 0.326 | 1.0000 |
| Reaming ⌀ 14.5 mm | | Reaming ⌀ 15.5 mm | | | -0.2000 | 0.327 | 1.0000 |
| Reaming ⌀ 14.5 mm | | Completion surgery +5 min | | | 0.0167 | 0.312 | 1.0000 |
| Reaming ⌀ 15 mm | | Reaming ⌀ 15.5 mm | | | 0.0000 | 0.326 | 1.0000 |
| Reaming ⌀ 15 mm | | Completion surgery +5 min | | | 0.2167 | 0.312 | 1.0000 |
| Reaming ⌀ 15.5 mm | | Completion surgery +5 min | | | 0.2167 | 0.312 | 1.0000 |
| **A+R+A group** | | | | | | | |
| **Time points** | | | | | **Estimated mean difference** | **Standard error** | **P - value** |
| Before surgery (Baseline) | | Opening femur | | | 0.1000 | 0.325 | 1.0000 |
| Before surgery (Baseline) | | Harvesting bone marrow | | | -0.0333 | 0.312 | 1.0000 |
| Before surgery (Baseline) | | Reaming ⌀ 10 mm | | | -0.0750 | 0.346 | 1.0000 |
| Before surgery (Baseline) | | Reaming ⌀ 10.5 mm | | | 0.0500 | 0.434 | 1.0000 |
| Before surgery (Baseline) | | Reaming ⌀ 11 mm | | | -0.2000 | 0.346 | 1.0000 |
| Before surgery (Baseline) | | Reaming ⌀ 11.5 mm | | | 0.3000 | 0.570 | 1.0000 |
| Before surgery (Baseline) | | Reaming ⌀ 12 mm | | | -0.2000 | 0.346 | 1.0000 |
| Before surgery (Baseline) | | Reaming ⌀ 12.5 mm | | | 0.3000 | 0.325 | 0.9999 |
| Before surgery (Baseline) | | Reaming ⌀ 13 mm | | | 0.3000 | 0.326 | 0.9999 |
| Before surgery (Baseline) | | Reaming ⌀ 13.5 mm | | | 0.1000 | 0.325 | 1.0000 |
| Before surgery (Baseline) | | Reaming ⌀ 14 mm | | | -0.1000 | 0.326 | 1.0000 |
| Before surgery (Baseline) | | Reaming ⌀ 14.5 mm | | | 0.0500 | 0.312 | 1.0000 |
| Before surgery (Baseline) | | Reaming ⌀ 15 mm | | | 0.3000 | 0.346 | 1.0000 |
| Before surgery (Baseline) | | Reaming ⌀ 15.5 mm | | | 0.3000 | 0.377 | 1.0000 |
| Before surgery (Baseline) | | Completion surgery +5 min | | | 0.0500 | 0.312 | 1.0000 |
| Opening femur | | Harvesting bone marrow | | | -0.1333 | 0.312 | 1.0000 |
| Opening femur | | Reaming ⌀ 10 mm | | | -0.1750 | 0.346 | 1.0000 |
| Opening femur | | Reaming ⌀ 10.5 mm | | | -0.0500 | 0.434 | 1.0000 |
| Opening femur | | Reaming ⌀ 11 mm | | | -0.3000 | 0.346 | 1.0000 |
| Opening femur | | Reaming ⌀ 11.5 mm | | | 0.2000 | 0.570 | 1.0000 |
| Opening femur | | Reaming ⌀ 12 mm | | | -0.3000 | 0.346 | 1.0000 |
| Opening femur | | Reaming ⌀ 12.5 mm | | | 0.2000 | 0.325 | 1.0000 |
| Opening femur | | Reaming ⌀ 13 mm | | | 0.2000 | 0.326 | 1.0000 |
| Opening femur | | Reaming ⌀ 13.5 mm | | | 0.0000 | 0.325 | 1.0000 |
| Opening femur | | Reaming ⌀ 14 mm | | | -0.2000 | 0.326 | 1.0000 |
| Opening femur | | Reaming ⌀ 14.5 mm | | | -0.0500 | 0.312 | 1.0000 |
| Opening femur | | Reaming ⌀ 15 mm | | | 0.2000 | 0.346 | 1.0000 |
| Opening femur | | Reaming ⌀ 15.5 mm | | | 0.2000 | 0.377 | 1.0000 |
| Opening femur | | Completion surgery +5 min | | | -0.0500 | 0.312 | 1.0000 |
| Harvesting bone marrow | | Reaming ⌀ 10 mm | | | -0.0417 | 0.333 | 1.0000 |
| Harvesting bone marrow | | Reaming ⌀ 10.5 mm | | | 0.0833 | 0.423 | 1.0000 |
| Harvesting bone marrow | | Reaming ⌀ 11 mm | | | -0.1667 | 0.333 | 1.0000 |
| Harvesting bone marrow | | Reaming ⌀ 11.5 mm | | | 0.3333 | 0.563 | 1.0000 |
| Harvesting bone marrow | | Reaming ⌀ 12 mm | | | -0.1667 | 0.333 | 1.0000 |
| Harvesting bone marrow | | Reaming ⌀ 12.5 mm | | | 0.3333 | 0.312 | 0.9994 |
| Harvesting bone marrow | | Reaming ⌀ 13 mm | | | 0.3333 | 0.312 | 0.9994 |
| Harvesting bone marrow | | Reaming ⌀ 13.5 mm | | | 0.1333 | 0.312 | 1.0000 |
| Harvesting bone marrow | | Reaming ⌀ 14 mm | | | -0.0667 | 0.312 | 1.0000 |
| Harvesting bone marrow | | Reaming ⌀ 14.5 mm | | | 0.0833 | 0.297 | 1.0000 |
| Harvesting bone marrow | | Reaming ⌀ 15 mm | | | 0.3333 | 0.333 | 0.9997 |
| Harvesting bone marrow | | Reaming ⌀ 15.5 mm | | | 0.3333 | 0.366 | 0.9999 |
| Harvesting bone marrow | | Completion surgery +5 min | | | 0.0833 | 0.297 | 1.0000 |
| Reaming ⌀ 10 mm | | Reaming ⌀ 10.5 mm | | | 0.1250 | 0.452 | 1.0000 |
| Reaming ⌀ 10 mm | | Reaming ⌀ 11 mm | | | -0.1250 | 0.364 | 1.0000 |
| Reaming ⌀ 10 mm | | Reaming ⌀ 11.5 mm | | | 0.3750 | 0.586 | 1.0000 |
| Reaming ⌀ 10 mm | | Reaming ⌀ 12 mm | | | -0.1250 | 0.364 | 1.0000 |
| Reaming ⌀ 10 mm | | Reaming ⌀ 12.5 mm | | | 0.3750 | 0.346 | 0.9993 |
| Reaming ⌀ 10 mm | | Reaming ⌀ 13 mm | | | 0.3750 | 0.347 | 0.9994 |
| Reaming ⌀ 10 mm | | Reaming ⌀ 13.5 mm | | | 0.1750 | 0.346 | 1.0000 |
| Reaming ⌀ 10 mm | | Reaming ⌀ 14 mm | | | -0.0250 | 0.347 | 1.0000 |
| Reaming ⌀ 10 mm | | Reaming ⌀ 14.5 mm | | | 0.1250 | 0.333 | 1.0000 |
| Reaming ⌀ 10 mm | | Reaming ⌀ 15 mm | | | 0.3750 | 0.365 | 0.9997 |
| Reaming ⌀ 10 mm | | Reaming ⌀ 15.5 mm | | | 0.3750 | 0.396 | 0.9999 |
| Reaming ⌀ 10 mm | | Completion surgery +5 min | | | 0.1250 | 0.333 | 1.0000 |
| Reaming ⌀ 10.5 mm | | Reaming ⌀ 11 mm | | | -0.2500 | 0.452 | 1.0000 |
| Reaming ⌀ 10.5 mm | | Reaming ⌀ 11.5 mm | | | 0.2500 | 0.633 | 1.0000 |
| Reaming ⌀ 10.5 mm | | Reaming ⌀ 12 mm | | | -0.2500 | 0.452 | 1.0000 |
| Reaming ⌀ 10.5 mm | | Reaming ⌀ 12.5 mm | | | 0.2500 | 0.434 | 1.0000 |
| Reaming ⌀ 10.5 mm | | Reaming ⌀ 13 mm | | | 0.2500 | 0.433 | 1.0000 |
| Reaming ⌀ 10.5 mm | | Reaming ⌀ 13.5 mm | | | 0.0500 | 0.434 | 1.0000 |
| Reaming ⌀ 10.5 mm | | Reaming ⌀ 14 mm | | | -0.1500 | 0.433 | 1.0000 |
| Reaming ⌀ 10.5 mm | | Reaming ⌀ 14.5 mm | | | 0.0000 | 0.423 | 1.0000 |
| Reaming ⌀ 10.5 mm | | Reaming ⌀ 15 mm | | | 0.2500 | 0.449 | 1.0000 |
| Reaming ⌀ 10.5 mm | | Reaming ⌀ 15.5 mm | | | 0.2500 | 0.473 | 1.0000 |
| Reaming ⌀ 10.5 mm | | Completion surgery +5 min | | | 0.0000 | 0.423 | 1.0000 |
| Reaming ⌀ 11 mm | | Reaming ⌀ 11.5 mm | | | 0.5000 | 0.586 | 1.0000 |
| Reaming ⌀ 11 mm | | Reaming ⌀ 12 mm | | | 0.0000 | 0.364 | 1.0000 |
| Reaming ⌀ 11 mm | | Reaming ⌀ 12.5 mm | | | 0.5000 | 0.346 | 0.9859 |
| Reaming ⌀ 11 mm | | Reaming ⌀ 13 mm | | | 0.5000 | 0.347 | 0.9863 |
| Reaming ⌀ 11 mm | | Reaming ⌀ 13.5 mm | | | 0.3000 | 0.346 | 1.0000 |
| Reaming ⌀ 11 mm | | Reaming ⌀ 14 mm | | | 0.1000 | 0.347 | 1.0000 |
| Reaming ⌀ 11 mm | | Reaming ⌀ 14.5 mm | | | 0.2500 | 0.333 | 1.0000 |
| Reaming ⌀ 11 mm | | Reaming ⌀ 15 mm | | | 0.5000 | 0.365 | 0.9917 |
| Reaming ⌀ 11 mm | | Reaming ⌀ 15.5 mm | | | 0.5000 | 0.396 | 0.9964 |
| Reaming ⌀ 11 mm | | Completion surgery +5 min | | | 0.2500 | 0.333 | 1.0000 |
| Reaming ⌀ 11.5 mm | | Reaming ⌀ 12 mm | | | -0.5000 | 0.586 | 1.0000 |
| Reaming ⌀ 11.5 mm | | Reaming ⌀ 12.5 mm | | | 0.0000 | 0.570 | 1.0000 |
| Reaming ⌀ 11.5 mm | | Reaming ⌀ 13 mm | | | 0.0000 | 0.570 | 1.0000 |
| Reaming ⌀ 11.5 mm | | Reaming ⌀ 13.5 mm | | | -0.2000 | 0.570 | 1.0000 |
| Reaming ⌀ 11.5 mm | | Reaming ⌀ 14 mm | | | -0.4000 | 0.570 | 1.0000 |
| Reaming ⌀ 11.5 mm | | Reaming ⌀ 14.5 mm | | | -0.2500 | 0.563 | 1.0000 |
| Reaming ⌀ 11.5 mm | | Reaming ⌀ 15 mm | | | 0.0000 | 0.582 | 1.0000 |
| Reaming ⌀ 11.5 mm | | Reaming ⌀ 15.5 mm | | | 0.0000 | 0.600 | 1.0000 |
| Reaming ⌀ 11.5 mm | | Completion surgery +5 min | | | -0.2500 | 0.563 | 1.0000 |
| Reaming ⌀ 12 mm | | Reaming ⌀ 12.5 mm | | | 0.5000 | 0.346 | 0.9859 |
| Reaming ⌀ 12 mm | | Reaming ⌀ 13 mm | | | 0.5000 | 0.347 | 0.9863 |
| Reaming ⌀ 12 mm | | Reaming ⌀ 13.5 mm | | | 0.3000 | 0.346 | 1.0000 |
| Reaming ⌀ 12 mm | | Reaming ⌀ 14 mm | | | 0.1000 | 0.347 | 1.0000 |
| Reaming ⌀ 12 mm | | Reaming ⌀ 14.5 mm | | | 0.2500 | 0.333 | 1.0000 |
| Reaming ⌀ 12 mm | | Reaming ⌀ 15 mm | | | 0.5000 | 0.365 | 0.9917 |
| Reaming ⌀ 12 mm | | Reaming ⌀ 15.5 mm | | | 0.5000 | 0.396 | 0.9964 |
| Reaming ⌀ 12 mm | | Completion surgery +5 min | | | 0.2500 | 0.333 | 1.0000 |
| Reaming ⌀ 12.5 mm | | Reaming ⌀ 13 mm | | | 0.0000 | 0.326 | 1.0000 |
| Reaming ⌀ 12.5 mm | | Reaming ⌀ 13.5 mm | | | -0.2000 | 0.325 | 1.0000 |
| Reaming ⌀ 12.5 mm | | Reaming ⌀ 14 mm | | | -0.4000 | 0.326 | 0.9974 |
| Reaming ⌀ 12.5 mm | | Reaming ⌀ 14.5 mm | | | -0.2500 | 0.312 | 1.0000 |
| Reaming ⌀ 12.5 mm | | Reaming ⌀ 15 mm | | | 0.0000 | 0.346 | 1.0000 |
| Reaming ⌀ 12.5 mm | | Reaming ⌀ 15.5 mm | | | 0.0000 | 0.377 | 1.0000 |
| Reaming ⌀ 12.5 mm | | Completion surgery +5 min | | | -0.2500 | 0.312 | 1.0000 |
| Reaming ⌀ 13 mm | | Reaming ⌀ 13.5 mm | | | -0.2000 | 0.326 | 1.0000 |
| Reaming ⌀ 13 mm | | Reaming ⌀ 14 mm | | | -0.4000 | 0.325 | 0.9973 |
| Reaming ⌀ 13 mm | | Reaming ⌀ 14.5 mm | | | -0.2500 | 0.312 | 1.0000 |
| Reaming ⌀ 13 mm | | Reaming ⌀ 15 mm | | | 0.0000 | 0.345 | 1.0000 |
| Reaming ⌀ 13 mm | | Reaming ⌀ 15.5 mm | | | 0.0000 | 0.377 | 1.0000 |
| Reaming ⌀ 13 mm | | Completion surgery +5 min | | | -0.2500 | 0.312 | 1.0000 |
| Reaming ⌀ 13.5 mm | | Reaming ⌀ 14 mm | | | -0.2000 | 0.326 | 1.0000 |
| Reaming ⌀ 13.5 mm | | Reaming ⌀ 14.5 mm | | | -0.0500 | 0.312 | 1.0000 |
| Reaming ⌀ 13.5 mm | | Reaming ⌀ 15 mm | | | 0.2000 | 0.346 | 1.0000 |
| Reaming ⌀ 13.5 mm | | Reaming ⌀ 15.5 mm | | | 0.2000 | 0.377 | 1.0000 |
| Reaming ⌀ 13.5 mm | | Completion surgery +5 min | | | -0.0500 | 0.312 | 1.0000 |
| Reaming ⌀ 14 mm | | Reaming ⌀ 14.5 mm | | | 0.1500 | 0.312 | 1.0000 |
| Reaming ⌀ 14 mm | | Reaming ⌀ 15 mm | | | 0.4000 | 0.345 | 0.9986 |
| Reaming ⌀ 14 mm | | Reaming ⌀ 15.5 mm | | | 0.4000 | 0.377 | 0.9995 |
| Reaming ⌀ 14 mm | | Completion surgery +5 min | | | 0.1500 | 0.312 | 1.0000 |
| Reaming ⌀ 14.5 mm | | Reaming ⌀ 15 mm | | | 0.2500 | 0.333 | 1.0000 |
| Reaming ⌀ 14.5 mm | | Reaming ⌀ 15.5 mm | | | 0.2500 | 0.366 | 1.0000 |
| Reaming ⌀ 14.5 mm | | Completion surgery +5 min | | | 0.0000 | 0.297 | 1.0000 |
| Reaming ⌀ 15 mm | | Reaming ⌀ 15.5 mm | | | 0.0000 | 0.394 | 1.0000 |
| Reaming ⌀ 15 mm | | Completion surgery +5 min | | | -0.2500 | 0.333 | 1.0000 |
| Reaming ⌀ 15.5 mm | | Completion surgery +5 min | | | -0.2500 | 0.366 | 1.0000 |

## Suppl. Table 3. Differences in D-dimer concentration between the different time points within each experimental group.

|  | **RIA 2 group** | | | **A+R+A group** | | |
| --- | --- | --- | --- | --- | --- | --- |
| **Time points** | **Estimated mean (standard error)** | | **95% Confidence interval (lower / upper)** | **Estimated mean (standard error)** | **95% confidence interval (lower / upper)** | |
| Before surgery (Baseline) | 59.1 (7.07) | | (44.3 / 73.9) | 41.3 (7.07) | (26.6 / 56.1) | |
| Completion surgery +5 min | 59.1 (7.07) | | (44.4 / 73.9) | 46.8 (7.07) | (32.1 / 61.6) | |
| Completion surgery +30 min | 60.3 (7.07) | | (45.5 / 75.0) | 50.1 (7.07) | (35.4 / 64.9) | |
| Completion surgery +120 min | 63.9 (7.07) | | (49.1 / 78.7) | 48.5 (7.07) | (33.7 / 63.3) | |
| Completion surgery +240 min | 63.9 (7.07) | | (49.1 / 78.7) | 50.8 (7.07) | (36.0 / 65.5) | |
| **RIA 2 group** | | | | | | |
| **Time points** | | | | **Estimated mean difference (standard error)** | | **p-value** |
| Before surgery (Baseline) | | Completion surgery +5 min | | -0.01741 (4.44) | | 1.0000 |
| Before surgery (Baseline) | | Completion surgery +30 min | | -1.17465 (4.44) | | 0.9989 |
| Before surgery (Baseline) | | Completion surgery +120 min | | -4.79916 (4.44) | | 0.8159 |
| Before surgery (Baseline) | | Completion surgery +240 min | | -4.79615 (4.44) | | 0.8162 |
| Completion surgery +5 min | | Completion surgery +30 min | | -1.15724 (4.44) | | 0.9990 |
| Completion surgery +5 min | | Completion surgery +120 min | | -4.78175 (4.44) | | 0.8179 |
| Completion surgery +5 min | | Completion surgery +240 min | | -4.77874 (4.44) | | 0.8182 |
| Completion surgery +30 min | | Completion surgery +120 min | | -3.62451 (4.44) | | 0.9247 |
| Completion surgery +30 min | | Completion surgery +240 min | | -3.62151 (4.44) | | 0.9249 |
| Completion surgery +120 min | | Completion surgery +240 min | | 0.00301 (4.44) | | 1.0000 |
| **A+R+A group** | | | | | | |
| **Time points** | | | | **Estimated mean difference (standard error)** | | **p-value** |
| Before surgery (Baseline) | | Completion surgery +5 min | | -5.49432 (4.44) | | 0.7301 |
| Before surgery (Baseline) | | Completion surgery +30 min | | -8.80919 (4.44) | | 0.2877 |
| Before surgery (Baseline) | | Completion surgery +120 min | | -7.17029 (4.44) | | 0.4950 |
| Before surgery (Baseline) | | Completion surgery +240 min | | -9.43730 (4.44) | | 0.2245 |
| Completion surgery +5 min | | Completion surgery +30 min | | -3.31486 (4.44) | | 0.9445 |
| Completion surgery +5 min | | Completion surgery +120 min | | -1.67596 (4.44) | | 0.9956 |
| Completion surgery +5 min | | Completion surgery +240 min | | -3.94297 (4.44) | | 0.9003 |
| Completion surgery +30 min | | Completion surgery +120 min | | 1.63890 (4.44) | | 0.9959 |
| Completion surgery +30 min | | Completion surgery +240 min | | -0.62811 (4.44) | | 0.9999 |
| Completion surgery +120 min | | Completion surgery +240 min | | -2.26701 (4.44) | | 0.9860 |

## Suppl. Table. 4. Percentage area covered with fat of each lung lobe between experimental groups or within a group.

|  | **RIA 2 group** | | | | **A+R+A group** | | **Comparison experimental (RIA 2/A+R+A) groups** | | |
| --- | --- | --- | --- | --- | --- | --- | --- | --- | --- |
| **Lung lobes** | **Estimated mean (standard error)** | | **95% Confidence interval (lower / upper)** | | **Estimated mean (standard error)** | **95% confidence interval (lower / upper)** | **Estimated mean difference (standard error** | | **p - value** |
| Acces-sory | 0.0000517  (0.0000567) | | (-0.0000641  / 0.000168) | | 0.0000790  (0.0000567) | (-0.00003680  / 0.000195) | -0.0000273  (0.0000802) | | 0.7355 |
| Left lower | 0.0000562  (0.0000567) | | (-0.0000597  / 0.000172) | | 0.0001620  (0.0000567) | (0.00004570  / 0.000277) | -0.0001050  (0.0000802) | | 0.1991 |
| Left upper | 0.0000444  (0.0000567) | | (-0.0000714  / 0.000160) | | 0.0001150  (0.0000567) | (-0.00000133  / 0.000230) | -0.0000701  (0.0000802) | | 0.3890 |
| Right lower | 0.0000815  (0.0000567) | | (-0.0000344  / 0.000197) | | 0.0001080  (0.0000567) | (-0.00000752  / 0.000224) | -0.0000268  (0.0000802) | | 0.7401 |
| Right middle | 0.0000814  (0.0000567) | | (-0.0000345  / 0.000197) | | 0.0000620  (0.0000567) | (-0.00005380  / 0.000178) | 0.0000193  (0.0000802) | | 0.8110 |
| Right upper | 0.0001060  (0.0000567) | | (-0.0000101  / 0.000222) | | 0.0000626  (0.0000567) | (-0.00005320  / 0.000178) | 0.0000431  (0.0000802) | | 0.5946 |
| **RIA 2 group** | | | | | | | | | |
| **Lung lobes** | | | | **Estimated mean difference**  **(standard error)** | | | | **p-value** | |
| Accessory | | Left lower | | -0.00000448 (0.00005020) | | | | 1.0000 | |
| Accessory | | Left upper | | 0.00000729 (0.00005020) | | | | 1.0000 | |
| Accessory | | Right lower | | -0.00002980 (0.00005020) | | | | 0.9912 | |
| Accessory | | Right middle | | -0.00002970 (0.00005020) | | | | 0.9914 | |
| Accessory | | Right upper | | -0.00005400 (0.00005020) | | | | 0.8894 | |
| Left lower | | Left upper | | 0.00001180 (0.00005020) | | | | 0.9999 | |
| Left lower | | Right lower | | -0.00002530 (0.00005020) | | | | 0.9959 | |
| Left lower | | Right middle | | -0.00002520 (0.00005020) | | | | 0.9960 | |
| Left lower | | Right upper | | -0.00004950 (0.00005020) | | | | 0.9208 | |
| Left upper | | Right lower | | -0.00003710 (0.00005020) | | | | 0.9765 | |
| Left upper | | Right middle | | -0.00003690 (0.00005020) | | | | 0.9768 | |
| Left upper | | Right upper | | -0.00006130 (0.00005020) | | | | 0.8253 | |
| Right lower | | Right middle | | 0.00000012 (0.00005020) | | | | 1.0000 | |
| Right lower | | Right upper | | -0.00002430 (0.00005020) | | | | 0.9966 | |
| Right middle | | Right upper | | -0.00002440 (0.00005020) | | | | 0.9965 | |
| **A+R+A group** | | | | | | | | | |
| **Lung lobes** | | | | **Estimated mean difference**  **(standard error)** | | | | **p-value** | |
| Accessory | | Left lower | | -0.00008250 (0.00005020) | | | | 0.5744 | |
| Accessory | | Left upper | | -0.00003550 (0.00005020) | | | | 0.9806 | |
| Accessory | | Right lower | | -0.00002930 (0.00005020) | | | | 0.9919 | |
| Accessory | | Right middle | | 0.00001700 (0.00005020) | | | | 0.9994 | |
| Accessory | | Right upper | | 0.00001640 (0.00005020) | | | | 0.9995 | |
| Left lower | | Left upper | | 0.00004700 (0.00005020) | | | | 0.9359 | |
| Left lower | | Right lower | | 0.00005320 (0.00005020) | | | | 0.8959 | |
| Left lower | | Right middle | | 0.00009950 (0.00005020) | | | | 0.3639 | |
| Left lower | | Right upper | | 0.00009890 (0.00005020) | | | | 0.3705 | |
| Left upper | | Right lower | | 0.00000619 (0.00005020) | | | | 1.0000 | |
| Left upper | | Right middle | | 0.00005250 (0.00005020) | | | | 0.9009 | |
| Left upper | | Right upper | | 0.00005190 (0.00005020) | | | | 0.9050 | |
| Right lower | | Right middle | | 0.00004630 (0.00005020) | | | | 0.9396 | |
| Right lower | | Right upper | | 0.00004570 (0.00005020) | | | | 0.9426 | |
| Right middle | | Right upper | | -0.00000058 (0.00005020) | | | | 1.0000 | |

## Suppl. Table 5. Average size of fat particles in individual lung lobes either between experimental groups or within a group.

|  | **RIA 2 group** | | | **A+R+A group** | | | **Comparison experimental (RIA 2/A+R+A) groups** | |
| --- | --- | --- | --- | --- | --- | --- | --- | --- |
| **Lung lobes** | **Estimated mean (standard error)** | **95% Confidence interval (lower / upper)** | | **Estimated mean (standard error)** | **95% confidence interval (lower / upper)** | | **Estimated mean difference (standard error)** | **P - value** |
| Accessory | 284 (76.5) | (132 / 436) | | 341 (76.5) | (189 / 493) | | -57.0126 (108) | 0.5996 |
| Left lower | 466 (76.5) | (314 / 618) | | 466 (76.5) | (314 / 618) | | 0.00168 (108) | 1.0000 |
| Left upper | 365 (76.5) | (213 / 518) | | 319 (76.5) | (167 / 471) | | 46.32819 (108) | 0.6696 |
| Right lower | 479 (76.5) | (327 / 631) | | 360 (76.5) | (208 / 512) | | 119.09394 (108) | 0.2741 |
| Right middle | 464 (76.5) | (312 / 617) | | 379 (76.5) | (227 / 531) | | 85.26904 (108) | 0.4328 |
| Right upper | 386 (76.5) | (234 / 538) | | 330 (76.5) | (178 / 482) | | 56.27355 (108) | 0.6043 |
| **RIA 2 group** | | | | | | | | |
| **Lung lobes** | | | **Estimated mean difference (standard error)** | | | **p-value** | | |
| Accessory | Left lower | | -181.74 (104) | | | 0.5074 | | |
| Accessory | Left upper | | -81.46 (104) | | | 0.9697 | | |
| Accessory | Right lower | | -194.75 (104) | | | 0.4290 | | |
| Accessory | Right middle | | -180.33 (104) | | | 0.5161 | | |
| Accessory | Right upper | | -102.21 (104) | | | 0.9224 | | |
| Left lower | Left upper | | 100.28 (104) | | | 0.9280 | | |
| Left lower | Right lower | | -13.01 (104) | | | 1.0000 | | |
| Left lower | Right middle | | 1.41 (104) | | | 1.0000 | | |
| Left lower | Right upper | | 79.53 (104) | | | 0.9727 | | |
| Left upper | Right lower | | -113.29 (104) | | | 0.8846 | | |
| Left upper | Right middle | | -98.87 (104) | | | 0.9320 | | |
| Left upper | Right upper | | -20.75 (104) | | | 1.0000 | | |
| Right lower | Right middle | | 14.42 (104) | | | 1.0000 | | |
| Right lower | Right upper | | 92.54 (104) | | | 0.9480 | | |
| Right middle | Right upper | | 78.12 (104) | | | 0.9747 | | |
| **A+R+A group** | | | | | | | | |
| **Lung lobes** | | | **Estimated mean difference (standard error)** | | | **p-value** | | |
| Accessory | Left lower | | -124.73 (104) | | | 0.8366 | | |
| Accessory | Left upper | | 21.88 (104) | | | 0.9999 | | |
| Accessory | Right lower | | -18.65 (104) | | | 1.0000 | | |
| Accessory | Right middle | | -38.05 (104) | | | 0.9991 | | |
| Accessory | Right upper | | 11.07 (104) | | | 1.0000 | | |
| Left lower | Left upper | | 146.61 (104) | | | 0.7223 | | |
| Left lower | Right lower | | 106.08 (104) | | | 0.9102 | | |
| Left lower | Right middle | | 86.68 (104) | | | 0.9605 | | |
| Left lower | Right upper | | 135.80 (104) | | | 0.7819 | | |
| Left upper | Right lower | | -40.53 (104) | | | 0.9988 | | |
| Left upper | Right middle | | -59.93 (104) | | | 0.9923 | | |
| Left upper | Right upper | | -10.81 (104) | | | 1.0000 | | |
| Right lower | Right middle | | -19.40 (104) | | | 1.0000 | | |
| Right lower | Right upper | | 29.72 (104) | | | 0.9997 | | |
| Right middle | Right upper | | 49.12 (104) | | | 0.9970 | | |

## Suppl. Table 6. Average number of fat particles in individual lung lobes either between experimental groups or within a group.

|  | **RIA 2 group** | | | | **A+R+A group** | | | **Comparison experimental (RIA 2/A+R+A) groups** | |
| --- | --- | --- | --- | --- | --- | --- | --- | --- | --- |
| **Lung lobes** | **Estimated mean (standard error)** | | **95% Confidence interval (lower / upper)** | | **Estimated mean (standard error)** | **95% confidence interval (lower / upper)** | | **Estimated mean difference (standard error)** | **P - value** |
| Accessory | | 11.96 (8.25) | (-4.901 / 28.8) | | 16.12 (8.25) | (-0.735 / 33.0) | | -4.17 (11.7) | 0.7235 |
| Left lower | | 11.21 (8.25) | (-5.651 / 28.1) | | 19.88 (8.25) | (3.015 / 36.7) | | -8.67 (11.7) | 0.4633 |
| Left upper | | 6.75 (8.25) | (-10.110 / 23.6) | | 10.42 (8.25) | (-6.443 / 27.3) | | -3.67 (11.7) | 0.7555 |
| Right lower | | 12.33 (8.25) | (-4.526 / 29.2) | | 19.08 (8.25) | (2.224 / 35.9) | | -6.75 (11.7) | 0.5672 |
| Right middle | | 17.17 (8.25) | (0.307 / 34.0) | | 12.67 (8.25) | (-4.193 / 29.5) | | 4.50 (11.7) | 0.7024 |
| Right upper | | 16.17 (8.25) | (-0.693 / 33.0) | | 12.42 (8.25) | (-4.443 / 29.3) | | 3.75 (11.7) | 0.7501 |
| **RIA 2 group** | | | | | | | | | |
| **Lung lobes** | | | | **Estimated mean difference (standard error)** | | | **p-value** | | |
| Accessory | | Left lower | | 0.750 (7.27) | | | 1.0000 | | |
| Accessory | | Left upper | | 5.208 (7.27) | | | 0.9793 | | |
| Accessory | | Right lower | | -0.375 (7.27) | | | 1.0000 | | |
| Accessory | | Right middle | | -5.208 (7.27) | | | 0.9793 | | |
| Accessory | | Right upper | | -4.208 (7.27) | | | 0.9921 | | |
| Left lower | | Left upper | | 4.458 (7.27) | | | 0.9897 | | |
| Left lower | | Right lower | | -1.125 (7.27) | | | 1.0000 | | |
| Left lower | | Right middle | | -5.958 (7.27) | | | 0.9630 | | |
| Left lower | | Right upper | | -4.958 (7.27) | | | 0.9834 | | |
| Left upper | | Right lower | | -5.583 (7.27) | | | 0.9720 | | |
| Left upper | | Right middle | | -10.417 (7.27) | | | 0.7072 | | |
| Left upper | | Right upper | | -9.417 (7.27) | | | 0.7867 | | |
| Right lower | | Right middle | | -4.833 (7.27) | | | 0.9852 | | |
| Right lower | | Right upper | | -3.833 (7.27) | | | 0.9949 | | |
| Right middle | | Right upper | | 1.000 (7.27) | | | 1.0000 | | |
| **A+R+A group** | | | | | | | | | |
| **Lung lobes** | | | | **Estimated mean difference (standard error)** | | | **p-value** | | |
| Accessory | | Left lower | | -3.750 (7.27) | | | 0.9954 | | |
| Accessory | | Left upper | | 5.708 (7.27) | | | 0.9692 | | |
| Accessory | | Right lower | | -2.958 (7.27) | | | 0.9985 | | |
| Accessory | | Right middle | | 3.458 (7.27) | | | 0.9969 | | |
| Accessory | | Right upper | | 3.708 (7.27) | | | 0.9956 | | |
| Left lower | | Left upper | | 9.458 (7.27) | | | 0.7836 | | |
| Left lower | | Right lower | | 0.792 (7.27) | | | 1.0000 | | |
| Left lower | | Right middle | | 7.208 (7.27) | | | 0.9192 | | |
| Left lower | | Right upper | | 7.458 (7.27) | | | 0.9077 | | |
| Left upper | | Right lower | | -8.667 (7.27) | | | 0.8393 | | |
| Left upper | | Right middle | | -2.250 (7.27) | | | 0.9996 | | |
| Left upper | | Right upper | | -2.000 (7.27) | | | 0.9998 | | |
| Right lower | | Right middle | | 6.417 (7.27) | | | 0.9495 | | |
| Right lower | | Right upper | | 6.667 (7.27) | | | 0.9409 | | |
| Right middle | | Right upper | | 0.250 (7.27) | | | 1.0000 | | |

# References

1 Laubach M, Bessot A, McGovern J, et al. (2023) An in vivo study to investigate an original intramedullary bone graft harvesting technology. European Journal of Medical Research, 28(1):349

2 Ridler T (1978) Picture thresholding using an iterative selection method. IEEE Transactions on Systems, Man, and Cybernetics, 8:630-632

3 Shelley DA, Sih BL, Ng LJ (2014) An integrated physiology model to study regional lung damage effects and the physiologic response. Theor Biol Med Model, 11:32-32

4 Sparks DS, Saifzadeh S, Savi FM, et al. (2020) A preclinical large-animal model for the assessment of critical-size load-bearing bone defect reconstruction. Nat Protoc, 15(3):877-924

5 Stürmer KM (1993) Measurement of intramedullary pressure in an animal experiment and propositions to reduce the pressure increase. Injury, 24:S7-S21

6 Stürmer KM, Schuchardt W (1980) [New aspects of closed intramedullary nailing and marrow cavity reaming in animal experiments. II. Intramedullary pressure in marrow cavity reaming (author's transl)]. Unfallheilkunde, 83(7):346-352
